# Circulating Endothelial Progenitor Cells in Castration Resistant Prostate Cancer: A Randomized, Controlled, Biomarker Study

**DOI:** 10.1371/journal.pone.0095310

**Published:** 2014-04-22

**Authors:** Thorsten Fuereder, Volker Wacheck, Sabine Strommer, Peter Horak, Marion Gerschpacher, Wolfgang Lamm, Danijel Kivaranovic, Michael Krainer

**Affiliations:** 1 Department of Internal Medicine I, Medical University of Vienna, Vienna, Austria; 2 Department of Clinical Pharmacology, Medical University of Vienna, Vienna, Austria; 3 Section for Medical Statistics, Medical University of Vienna, Vienna, Austria; University Clinic of Navarra, Spain

## Abstract

**Background:**

Endothelial progenitor cells (CEPs) and circulating endothelial cells (CECs) are potential biomarkers of response to anti-angiogenic treatment regimens. In the current study, we investigated the effect of docetaxel and sunitinib on CEP/CEC kinetics and clinical response in castration resistant prostate cancer (CRPC) patients.

**Patients and methods:**

Chemonaive patients with CRPC were enrolled in this study to receive either sunitinib (37.5 mg/d), in combination with docetaxel (75 mg/m^2^) or docetaxel alone. CEP and CEC kinetics were analyzed for every cycle. The primary objective was to compare CEP/CEC pharmacodynamics between both treatment arms. We also investigated if CEC/CEP spikes, induced by MTD docetaxel, are suppressed by sunitinib in patients treated with docetaxel/sunitinib relative to docetaxel monotherapy.

**Results:**

A total of 27 patients were enrolled. We observed a significant increase of CEP/CEC (total/viable) counts over time within each cycle (coefficients 0.29233, 0.22092 and 0.26089, respectively; p<0.001). However, no differences between the treatment groups, in terms of CEP and CEC kinetics, were detected. In the docetaxel monotherapy arm 4 (30%) patients responded to therapy with a 50% PSA decline, while 9 (64%) patients showed a PSA decline in the combination group (n.s.). The median PFS in the docetaxel monotherapy group was 3.1 months (2.6–3.6 months, 95% CI) and 6.2 months (4.9–7.4 months, 95% CI; p = 0.062) in the combination arm. Sunitinib/docetaxel was reasonably well tolerated and toxicity manageable.

**Conclusion:**

In summary, no significant differences in CEC and CEP kinetics between the treatment arms were observed, although a highly significant increase of CEPs/CECs within each cycle over time was detected. These results mirror the challenge we have to face when employing anti-angiogenic strategies in CRPC. Additional preclinical research is needed to elucidate the underlying molecular mechanisms. However, docetaxel/sunitinib therapy resulted in a better response in terms of PSA decline and a trend towards improved PFS.

**Trial Registery:**

clinicaltrialsregister.eu EudraCT 2007-003705-27

## Introduction

Prostate cancer is the most common cancer among males, and accounts for the second most common cause of cancer related deaths [1]. Based on two landmark trials docetaxel was considered the standard of care in CRPC for many years [2], [3]. Very recently, three novel compounds, abiraterone, enzalutamide and cabazitaxel, were approved for CRPC treatment [4], [5]. Sipuleucel–T provides an additional treatment option [6]. Although these novel therapeutics resulted in a significant increase in prostate cancer survival, there is still an urgent need for novel treatment concepts. The tumor vasculature has emerged as a clinically validated therapeutic target since the 1970s [7]. Intensive research resulted in a better understanding of the mechanisms of angiogenesis. CEPs, a small subpopulation of bone marrow derived endothelial cells, were identified as pivotal contributors to the process of *de novo* vasculogenesis [8]. Additionally, CECs were investigated and found to be elevated in human malignancies [8]. There is extensive evidence that both conventional chemotherapy and anti-angiogenic drugs modulate CEP and CEC kinetics reflecting both, a response to anti-angiogenic therapy, and an escape mechanism to cytotoxic chemotherapy [8], [9]. In particular, administration of chemotherapy at the maximum tolerated dose can lead to CEP recruitment, which home to the viable tumor rim that characteristically remains after such therapy [10]. Disruption of the CEP spike by anti-angiogenic drugs resulted in a marked reduction in tumor rim size and blood flow *in vivo* [10].To date, several inhibitors of angiogenesis have been approved for the treatment of human malignancies. Amongst them sunitinib is extensively studied in clinical trials and is approved for renal cell cancer, GIST and neuroendocrine pancreatic cancer. However for CRPC, the initial promising results of preclinical anti-angiogenic approaches, testing both anti-angiogenic small molecule VEGFR inhibitors and antibodies, could not be translated into the clinic. For instance, sunitinib monotherapy, which was evaluated in a large multicentre phase III study, did not meet the primary endpoint of prolonged overall survival, despite an increase in PFS [11]–[13]. Due to complex alterations in the tumor’s genetic background, targeting the tumor vasculature by anti-angiogenic monotherapy is not sufficient in most cases. Thus, the vast majority of anti-angiogenic compounds, employed in the clinic, are implemented in the form of combination strategies. Consistently, there is good preclinical evidence that combination strategies of sunitinib with conventional chemotherapeutic drugs are beneficial [14]. Based on this background we hypothesized that sunitinib therapy of CRPC patients can blunt docetaxel induced CEP spikes, which should result in a better clinical outcome when compared to docetaxel monotherapy. Additionally, we sought to get better insight into the mechanisms for anti-angiogenic treatment failure, which has been observed in CRPC patients so far. Here, we report the results of an exploratory biomarker trial investigating CEP and CEC kinetics in CRPC patients treated with sunitinib/docetaxel versus docetaxel monotherapy.

## Patients and Methods

The protocol for this trial and supporting CONSORT checklist are available as supporting information; see Checklist S1 and Protocol S1.

### Study Design and Patient Selection

The primary objective of this exploratory, 1∶1 randomized, controlled, open-label study was to determine whether the expected CEC/CEP spikes, induced by docetaxel, are suppressed by sunitinib in chemonaive CRPC patients treated either with docetaxel/sunitinib or docetaxel monotherapy. In addition, we aimed to compare CEP/CEC kinetics between the two treatment groups. Secondary objectives were (i) to assess whether docetaxel/sunitinib increases response rate and length of treatment holidays relative to docetaxel monotherapy, and (ii) whether treatment by sunitinib/docetaxel is safe and tolerable.

The selected patients suffered from histologically confirmed and advanced CRPC, were required to have an ECOG performance status of 0 to 2, and adequate organ function (bilirubin ≤1, 5×UNL (upper normal level), ASAT, ALAT ≤1.5×UNL, creatinine ≤1.5 UNL, WBC ≥3.5×109/L, ANC ≥1.5×109/L, Hb ≥10 g/dl, platelets ≥100×109/L). Additionally, LVEF measured by echocardiography had to be 50%, and patients with relevant cardiac disorders were excluded. Before recruitment, the objective and nature of the trial were explained to patients, who then had to sign an informed consent form.

A person not part of the study team generated the randomization list using a web based randomization plan generator (block size of four). Patient allocation envelopes were created according to the randomization list, and the envelopes were sealed, signed and dated by this person. The study nurse then assigned patients to interventions according to the patient allocation envelopes.

The study, which was conducted between 2008 and 2012, consisted of two consecutive treatment parts. In part I, all patients received standard care therapy of docetaxel (75 mg/m2 q 21d×4 cycles). Prednisolone (2×5 mg per day) was administered beginning from cycle 3. In addition to standard therapy, patients randomized in arm A received sunitinib (37.5 mg/d) on a three-week repeat schedule, with two weeks of daily treatment followed by a one-week rest starting the day after each docetaxel administration. Since early changes in CEC/CEP counts were expected, docetaxel treatment was stopped after four cycles. Patients were assessed by PSA and radiologic examination for response and sunitinib maintenance therapy, which should keep the CEP numbers low, was initiated for responders as described below.

Patients responding to treatment given in part I, as defined by a PSA decline of ≥50% compared to baseline, with no objective progression according to modRECIST, were eligible for part II of the trial [15]. All other patients (i.e. non-responders) were excluded from the study. Responders to docetaxel+sunitinib in part I were randomized either to sunitinib maintenance therapy (50 mg/d for 14 days followed by one week rest), or to no treatment. Responders to docetaxel received no treatment during their chemotherapy holidays. Treatment with or without sunitinib was prolonged as long as the patients had no PSA-progression (measured every three weeks) above the baseline. Whenever a patient progressed with a PSA above the baseline value, or a new radiologic lesion developed, his participation was terminated. The End of Study (EOS) visit was performed to follow-up on the adverse events, which were still ongoing during the last study visit. The EOS visit was conducted no earlier than 30 days after the last administration of the study medication. No additional follow-up visits were scheduled.

The trial was registered at the EU clinical trial register (EudraCT 2007-003705-27; https://www.clinicaltrialsregister.eu/ctr-search/search?query=2007-003705-27), and no patient was enrolled in the study before registration. Additionally, the study was registered at Clinical Trials.gov (NCT00795171; http://clinicaltrials.gov/ct2/show/NCT00795171?term=wacheck &rank = 4). The authors confirm that all ongoing and related trials for this drug/intervention are registered.

### Ethics Statement

The study was performed in accordance with the Declaration of Helsinki and good clinical practice guidelines, and was approved by the local ethics committee (the ethics committee of the Medical University of Vienna).

### CEP/CEC Assessment and Statistical Considerations

Quantification of CEC/CEP from peripheral blood (5 ml) by FACS analysis was performed according to a protocol provided by the Bertolini/Kerbel group in our institute [8], [16]. This consisted of the following: blood was collected in EDTA tubes and the first tube was discarded to prevent false positive CEC numbers resulting from vessel injury. Subsequently, FcR blocking reagent (Milteny Biotec, Germany) was added. Cells were then incubated and labeled with the following antibodies: For CEP enumeration, cells were stained with CD31 FITC (Becton Dickinson, USA, cat. number 555445), CD146PE (Merck Milipore, USA, cat. number MAB16985H), CD45PerCP (Becton Dickinson, USA, cat. number 345809) and CD133APC (Milteny Biotec, Germany, cat. number 130-090-826). For viable/dead CEC quantification, cells were labeled with CD31FITC (Becton Dickinson, USA), CD146PE (Merck Milipore, USA), CD45APC (Becton Dickinson, USA, cat. number 340910) and 7-AAD (Becton Dickinson, USA, cat. number 559925 (added briefly before measurement). For isotype controls, the following antibodies were employed: Mouse IgGk FITC (Becton Dickinson, USA, cat. number 5519954), mouse IgG1 APC (Milteny Biotec, Germany, cat. number 130-092-214) and mouse IgG FITC (Chemicon, USA, CBL600P). After incubation, washing and red blood cell lysis, CEP/CEC quantification was performed immediately using a FACSCalibur (Becton Dickinson, USA). At least 600,000 cells were acquired, and cells stained for CD133+; CD146+/CD45− and CD31+ were regarded as CEPs. Viable/dead CECs were 7AAD positive/negative and CD45−; CD146+, CD31+ and CD133−. After drawing the respective gates, a cut off for CD31 positivity was set at 4*10∧1 and at 10∧1 for 7AAD; CD146 and CD133. Settings were saved and used for every single measurement in the study to ensure comparability. Absolute CEP or CEC numbers were calculated as a percentage of positive cells on total events * white blood cell count.

Intra-subject variability was measured by determining CEP/CEC counts of healthy subjects in order to validate the assay. CEC/CEP kinetics were determined for each cycle at baseline, day 3 (i.e. ∼24 h after sunitinib administration), day 8 and day 15 (i.e. the end of sunitinib administration), immediately after blood sampling on the same day. Safety and tolerability were assessed by physical examinations and laboratory tests of patients during each of their visits, and toxicity was graded according to the Common Terminology Criteria for Adverse Events.

Initially, 60 patients were planned to be included in this trial. However, due to low recruitment rates and novel therapeutic options becoming available during the course of the trial, the study was terminated after recruiting 27 patients based on the following considerations:

According to the study by Tannock et al. [3], we expected about 50% of the patients to respond to docetaxel single therapy. Since the effect of sunitinib/docetaxel on CEC/CEP counts was unknown, and earlier studies reported that a significant difference in CEP/CEC kinetics could be detected in a small sample size, this sample size was considered sufficient for the PD-analysis of our primary objective. No additional benefit for meeting our primary objective was expected by recruiting additional patients. In addition, there was no further statistical justification for the sample size.

The changes in CEP, CEC total and CEC viable, within two treatment groups, group A and group B, were measured over time. Due to a large number of upward and downward outliers of the measured values, a logarithmic data transformation was used to maintain the conditions needed for meaningful hypothesis tests. The logarithmic data were used for subsequent analyses.

To analyze the difference in improvement between the two groups, linear mixed models were calculated. In the linear regression, the independent variables were the time within a cycle, and the interaction between time and the group. To consider the correlation of observations from the same patient, a random intercept term was included in the model. The computations were done using the lme4 package in R 3.0.2. The regressions were conducted for CEP, CEC total and CEC viable, separately. The significance level for all tests is 5%.

In order to test the equality of survivor functions between groups, the log-rank test was used. These statistical calculations were performed using SPSS 18.0 for Windows (SPSS Inc., Chicago, IL, USA).

## Results

### Patient Characteristics

**Figure 1:**
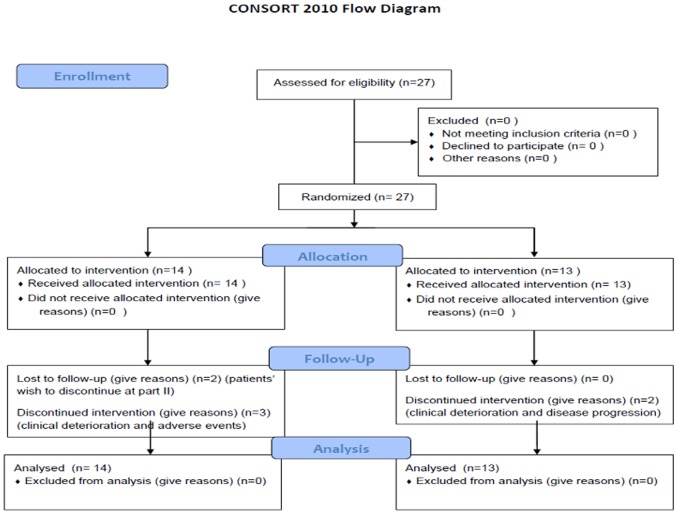
Consort flow diagram.

A total of 27 patients with CRPC were enrolled in the study between 2008 and 2012 (Table 1). 13 patients were randomized to docetaxel monotherapy group (arm B) and 14 patients to the docetaxel/sunitinib group (arm A) (Fig.1). Two patients in the docetaxel arm and three patients in the combination arm discontinued treatment prior to cycle 4 due to clinical deterioration, disease progression or grade 3/4 toxicity. The median age was 67 years (54–78) years in arm B, and 69 (58–77) years in arm A. Median PSA was 53 ng/dl (13.7–636.6) in arm B and 30 ng/dl (12.5–3350) in arm A. 53% of the patients in arm B had a Gleason score higher than 7 compared to 50% in arm A.

**Table 1:** Patient characteristics.

Characteristics	Docetaxel Monotherapy	Docetaxel/Sunitinib
Patient Number	13	14
Treated per protocol	11 (85%)	11 (79%)
Median age (range)	67 (54–78)	69 (58–77)
Race		
*White*	13 (100%)	13 (93%)
*Asian*	0 (0%)	1 (7%)
Disease Location		
*Bone*	10 (77%)	12 (85%)
*Node*	1 (8%)	3 (21%)
*Liver*	1 (8%)	0 (0%)
Gleason score		
≤7	4 (31%)	6 (43%)
8–10	7 (54%)	7 (50%)
Not available	2 (15%)	1 (7%)
Prostate-specific antigen		
*Median (range)*	53.1 ng/dl (13.7–636.6)	30.35 ng/dl (12.5–3350)
Treatment primary tumor		
*Surgery*	5 (38%)	7 (50%)
*Radiation*	3 (23%)	3 (21%)
*Other*	5 (38%)	4 (29%)
Prior hormone therapy		
*1 line*	4 (31%)	6 (43%)
*2 lines*	7 (54%)	5 (36%)
*3 lines*	2 (15%)	3 (21%)

### CEC and CEP Kinetics

**Figure 2:**
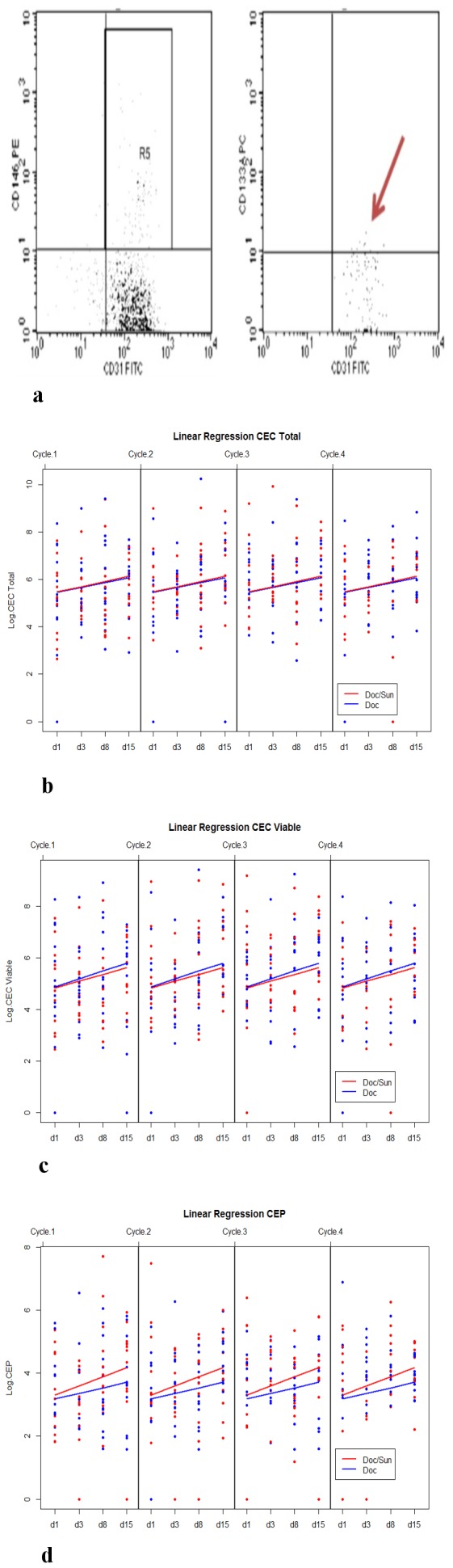
CEP and CEC kinetics. Representative example of flow cytometry dot plots chosen for CEP measurements. The left panel shows CD146 positive endothelial cells of which a small number were CD133 positive accounting for CEPs as indicated by the red arrow (right panel) (a). Regression analysis employing a linear mixed model of total CEC (b), viable CEC (c) and CEP (d) counts on a logarithmic scale of docetaxel (blue) and docetaxel/sunitinib treated patients. Each dot represents a single patient. X-axis represents cycles and time points; Y-axis represents CEP and CEC numbers on a logarithmic scale.

A linear mixed model was applied to test for significance in CEP and CEC kinetics. For CEPs and CECs (total and viable), a highly significant increase over time within each cycle was detected (coefficients 0.29233; 0.22092 and 0.26089 for CEPs, CECs total and CECs viable respectively; p<0.001). All three cell types namely the CEPs, viable CECs and total CECs were measured at the indicated time points (Fig. 2b–d). However, addition of sunitinib over time in each cycle resulted in no blunting or modulation in CEP or CEC kinetics.

During the maintenance period in part II of the study, median CEP counts slightly decreased until the time of progression (Fig. S1) in the docetaxel monotherapy group and the sunitinib maintenance group (0.8 fold median decrease). Withdrawal of sunitinib resulted in a non-significant (n.s.) 1.9-fold increase of CEP numbers. Similar results were observed for CEC kinetics (data not shown). A point to be noted is that we observed a high inter-patient variability both in total and viable CEC numbers and in CEP counts.

### PSA Response

**Figure 3:**
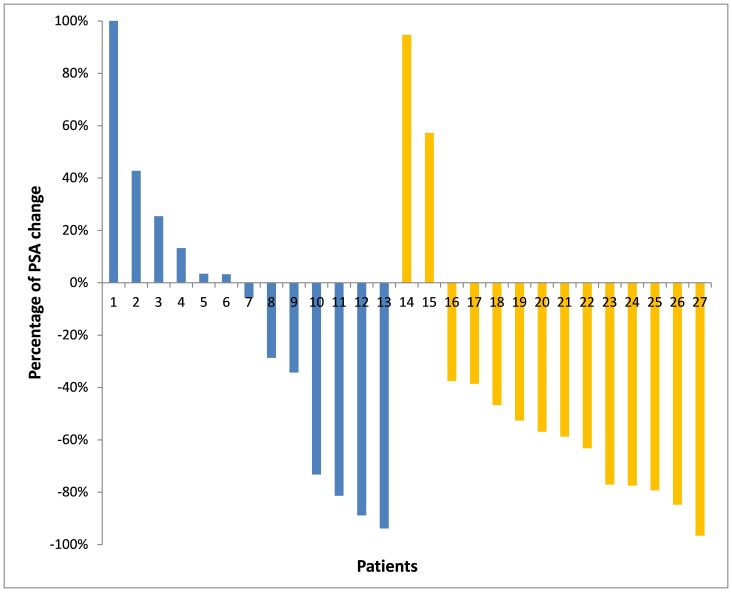
PSA response. Waterfall plot of PSA response to docetaxel (blue) and sunitinib/docetaxel (yellow) in CRPC patients.

PSA response is depicted as a waterfall plot in Figure 3 for each treatment arm. 5 (38%) patients in arm B and 12 (85%) patients in arm A had at least a 30% PSA reduction. A PSA decrease of 50% was considered as a response to therapy. In the docetaxel monotherapy arm (arm B) 4 (30%) patients responded to therapy, whereas 9 (64%) patients showed a PSA response in the combination group (n.s.). 3 patients (23%) in arm B and 8 (57%) patients in the combination arm (arm A) entered maintenance therapy according to the study protocol (Table 2). The median time of maintenance therapy until PSA progression (Table 3) was 2.6 (1.4–2.9) months in the docetaxel monotherapy group (no therapy), 2.6 (1.4–4.1) months in the sunitinib maintenance group and 2.1 (1.8–3.5) months in the sunitinib discontinuation group (no therapy).

**Table 2:** Bone scan results.

Bone scan	Docetaxel Monotherapy	Docetaxel/Sunitinib
No change or improvement	6 (46%)	8 (57%)
New lesions or increased tracer uptake	4 (30%)	1 (7%)
No bone lesions or N.A	3 (23%)	5 (36%)

**Table 3:** Part II patient characteristics.

	Docetaxel Monotherapy	Docetaxel/Sunitinib
Entered part II	3 (23%)	8 (57%)
Duration of treatment holidays or maintenance therapy	2.6 months (1.4–2.9)	Sunitib treatment
		2.6 months (1.4–4.1)
		Sunitinib discontinuation
		2.1 months (1.8–3.5)

### Bone Scan Measurements and Radiological Response

After 12 weeks of therapy radiological response, via computed tomography (CT), was assessed. Additionally bone scans were also performed (Table 3). 81% of the patients were evaluable for bone scan re-evaluation after 4 cycles of therapy. A decrease of tracer uptake or no change in bone lesions was observed in 8 (57%) patients in arm A and in 6 (47%) in arm B. 4 (30%) patients showed an increased tracer uptake in arm B and one patient (7%) in arm A. Only 5 (14%) patients in the study suffered from nodal or hepatic disease as detected by CT-scans. One partial response was observed in the docetaxel monotherapy group, and one stable disease was observed in the combination group. 3 patients were not assessable for CT-scan.

### Progression Free Survival

**Figure 4:**
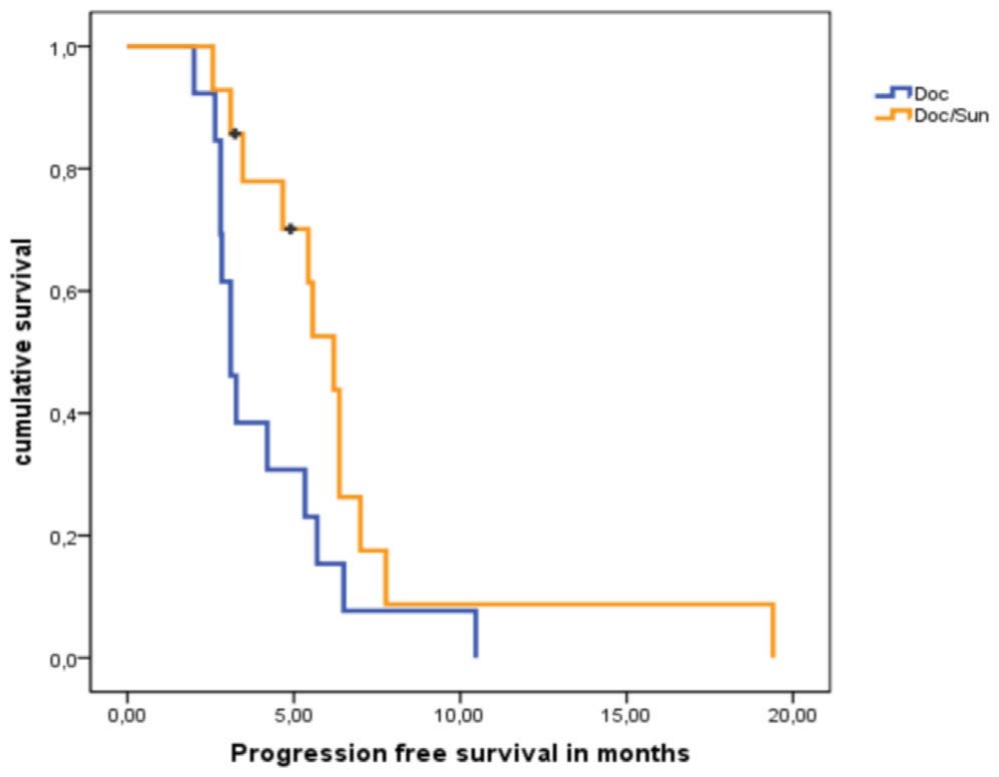
PFS between both treatment arms. Kaplan-Meier curves depicting progression free survival between sunitinib/docetaxel arm (orange) and docetaxel monotherapy arm (blue). Black bars represent censored patients.

25 patients were evaluated for PFS at the end of the trial (Fig. 4). Two patients in arm A wanted to discontinue and were lost to follow-up. The median PFS in the docetaxel monotherapy group was 3.1 months (2.6–3.6 months, 95% CI), whereas the median PFS in the combination arm was nearly twice (6.2 months; 4.9–7.4 95% CI). However, due to the small patient number this difference did not reach statistical significance (p = 0.062).

### Toxicity

In general, the combination of docetaxel and sunitinib was reasonably well-tolerated (Table S1). The most common adverse events were severe myelosuppression, which was comparable between the groups. All patients recovered quickly from neutropenia and could continue with the therapy. Toxicity in arm A was manageable and consisted of epistaxis, hypertension, nausea, diarrhea and two cases of elevated liver enzymes (all Grade 1 adverse events). One patient in arm A experienced episodes of recurrent premature ventricular contractions. The most severe grade 3 adverse events in the combination arm were one case of acute kidney failure and two cases of myocardial infarction.

## Discussion

Despite recent advances in the therapy of CRPC, there is still an unmet need of novel treatment strategies and rational combination regimens. In CRPC patients, anti-angiogenic strategies have not resulted in clinical benefit so far, and several anti-angiogenic trials failed to meet their clinical endpoint. Sunitinib combination strategies did not show superiority over standard therapy including colorectal cancer, breast cancer and CRPC in terms of survival advantage [17]–[19]. While dosing issues might have contributed to these disappointing results, it is obvious that the understanding of angiogenesis resistance mechanisms and the optimal employment of receptor tyrosine kinase inhibitors is still limited. Furthermore, a recent successful phase I/II sunitinib trial established the recommended phase II dose of sunitinib in combination with docetaxel. Based on these considerations, we chose this biomarker approach to investigate CEP and CEC kinetics in CRPC patients treated with sunitinib/docetaxel, to gain a better insight into the mechanisms of CRPC response to anti-angiogenic therapies. [19]. Our study found no significant modulation of total and viable CECs and CEPs following sunitinib/docetaxel therapy, although significant increases in their counts were observed over time within each cycle. First, we have to address the methodological considerations when interpreting our results. Although CEP and CEC counts found in this study are in accordance with other publications on cancer patients, there is no generally accepted definition of CEP and CEC surface markers [8], [20], [21]. The lack of an international consensus for CEP and CEC enumeration is one major challenge for further development of these biomarkers. Indeed there is no standard nomenclature or classification for CEPs or CECs. Reports characterizing a large proportion of CECs as immature platelets, myeloid cells or monocytes add to the complexity of this issue, which might have contributed to the conflicting results, reported both in pre-clinical and clinical CEP/CEC biomarker trials, to date [22]–[24].

In addition, we observed a considerable inter-patient variability in terms of CEP and CEC counts. Although this finding is in line with current literature, healthy subjects were measured on different days to test intra-patient variability and further validate the method [25]–[27]. These measurements yielded similar and reproducible CEC and CEP numbers on different days (data not shown). While inadequate power and low patient numbers might be a reason for the lack of modulation in CEC/CEP kinetics by sunitinib in this trial, we did not recruit additional patients, since novel treatment options became available for CRPC patients during the course of the trial. Given the limitations discussed above, we believe that both the distinct tumor biology of CRPC and the lack of well-defined CEP/CEC surface markers accounted for the results of this study for several reasons: Early preclinical reports demonstrated that CEPs and CECs play an important role in prostate cancer tumor biology and that cytotoxic or targeted therapy modulates CEP and CEC counts *in vitro* and *in vivo* [28]–[31]. In the clinical setting, however, the data available on CEP/CEC levels in prostate cancer patients are very limited. Moreover, clinical studies evaluating CEPs or CECs in prostate cancer patients show conflicting results. Some studies demonstrated a link between CEP/CEC levels and overall survival in CRPC patients treated with docetaxel, or with treatment response to bone directed therapies [32]–[34]. These studies used either multi-parametric FACS analysis or the Cell Tracks analyzer system for CEP/CEC evaluation, and either incorporated CD146, Syto6, CD133, CD105 and CD31 antibodies, or did not give any methodological details. In contrast, several (partly highly powered) clinical studies investigating both anti-angiogenic drugs and cytotoxic chemotherapies in prostate cancer patients showed no correlation between CEC or CEP levels with therapy response to other clinical endpoints [22], [35], [36]. These studies employed CD146, CD308, CD45 and CD34 antibodies for CEC/CEP quantification as measured again by multi-parametric FACS analysis or the Cell Tracks analyzer system. One of these studies did not report the detailed method for CEC analysis [35].

In general it has been demonstrated that CEC and CEP kinetics depend on the tumor type, the therapeutic regimen and the specific method for CEC/CEP quantification. An increase, a decrease, and even no change in CECs and CEP kinetics were detected following anti-angiogenic or cytotoxic treatment strategies [8], [18], [37].

Apart from the methodological considerations discussed, the reason for such a diverse behavior of CEP/CEC mobilization, both in prostate cancer and in other tumor types and clinical settings, remains unclear. In view of the negative anti-angiogenesis phase III trials including sunitinib monotherapy conducted in CRPC patients, our results might at least partially reflect a tumor escape mechanism to an anti-angiogenic treatment strategy in CRPC [13]. Anti-angiogenic drugs can elicit CEP mobilization from the bone marrow and foster adaptive mechanisms to overcome hypoxic challenges [9]. Finally, tumor independent mechanisms have to be taken into account. Although no data concerning CEC/CEP levels exist, previously described preclinical models show that sunitinib treatment induces tumor independent changes in multiple circulating pro-angiogenic factors [38].

A 50% PSA decline upon therapy is widely used in many clinical trials as a surrogate for response, while a ≥30% decline was demonstrated as a good predictor of clinical endpoints such as pain and overall survival [39]. Although discordant PSA and clinical responses were observed with tyrosine kinase inhibitors in prostate cancer patients, such a phenomenon seems to play a minor role for sunitinib therapy (but cannot be ruled out entirely) [12], [19]. We observed a response rate of 64% in the sunitinib combination group and 57% of the patients displayed an improvement or stabilization of bone lesions. Consistently, the PFS although statistically not significant, was prolonged in the combination arm. The median time of maintenance therapy until PSA progression was comparable in the no treatment and sunitinib maintenance groups. Given the limitations that this exploratory biomarker study was not designed or powered to prove superiority of sunitinib/docetaxel over docetaxel monotherapy, we must note that these data are in line with previous reports. These include studies that show sunitinib/docetaxel is beneficial, and that sunitinib monotherapy might not be sufficient to achieve long term disease control or survival advantage in CRPC patients who received docetaxel therapy [13], [19]. Although there are reports that the docetaxel/sunitinib regimen causes a higher frequency of adverse events (all grades), no new safety issues arose in our trial [17]. The observed toxicity profile of docetaxel in combination with sunitinib was similar to that reported in CRPC patients [19]. Two patients experienced myocardial infarction and had to discontinue the study. In general, the combination regimen was reasonably well tolerated.

In conclusion, CEP/CEC numbers were increased within each chemotherapy cycle in previously chemonaive CRPC patients treated with docetaxel/sunitinib or docetaxel alone. However, there is no significant difference in CEP and CEC kinetics between docetaxel monotherapy and in combination with sunitinib. These results reflect the challenge we have to face when employing anti-angiogenic treatment strategies in CRPC. Although the underlying molecular mechanisms remain unclear, CRPC patients responded to docetaxel/sunitinib combination in terms of PSA response and a trend towards improved PFS.

## Supporting Information

Figure S1 **Box plots of median x-fold change of CEP counts at the end of part II compared to the end of part I.**
(TIF)

Table S1 **Adverse events.**
(DOCX)

Checklist S1 **CONSORT 2010 checklist.**
(DOC)

Protocol S1 **Study protocol as approved by the local ethics committee.**
(PDF)
