# Supplementary figures and images for: Circulating Endothelial Progenitor Cells in Castration Resistant Prostate Cancer: A Randomized, Controlled, Biomarker Study
Source: PLoS One. 2014 Apr 22;9(4):e95310. doi: 10.1371/journal.pone.0095310 (PMC3995874; doi:10.1371/journal.pone.0095310)

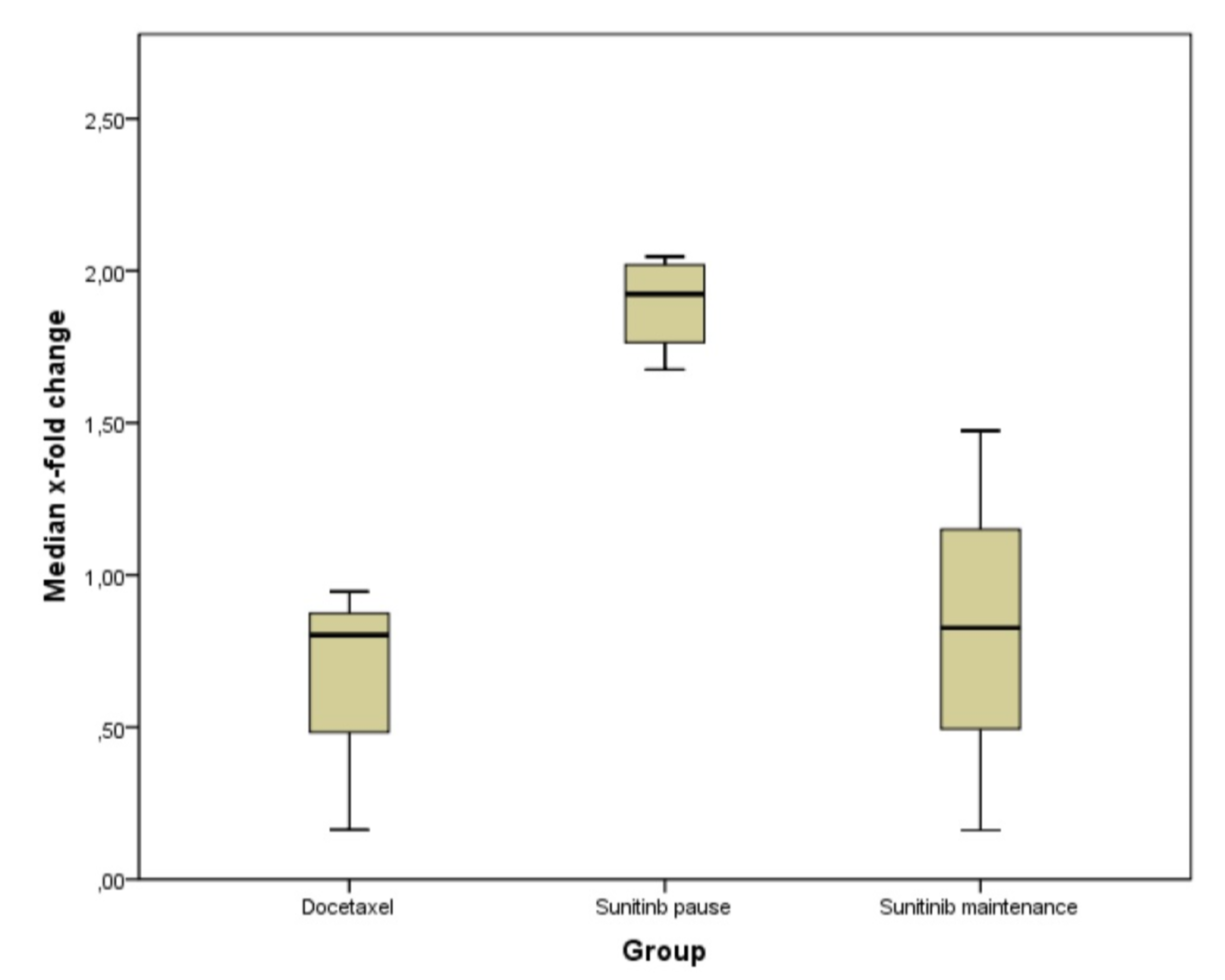

Supplement: Figure S1 — Box plots of median x-fold change of CEP counts at the end of part II compared to the end of part I. (TIF) [file pone.0095310.s001.tif]
