# Supplementary material for: Circulating Endothelial Progenitor Cells in Castration Resistant Prostate Cancer: A Randomized, Controlled, Biomarker Study
Source: PLoS One. 2014 Apr 22;9(4):e95310. doi: 10.1371/journal.pone.0095310 (PMC3995874; doi:10.1371/journal.pone.0095310)
Supplement: Table S1 — Adverse events. (DOCX) [file pone.0095310.s002.docx]

| **Event** | **Docetaxel Monotherapy**  **All grades** | **Docetaxel Monotherapy**  **Grade 3/4** | **Docetaxel/Sunitinib**  **All grades** | **Docetaxel/Sunitinib**  **Grade 3/4** |
| --- | --- | --- | --- | --- |
| **Anemia** | **13** | **0** | **14** | **3** |
| **Thrombopenia** | **1** | **0** | **2** | **0** |
| **Neutropenia** | **11** | **8** | **9** | **8** |
| **Fatigue** | **8** | **0** | **4** | **0** |
| **Diarrhea** | **1** | **0** | **8** | **0** |
| **Nausea** | **4** | **0** | **8** | **0** |
| **Dysgeusia** | **7** | **0** | **5** | **0** |
| **Fever** | **2** | **0** | **2** | **0** |
| **Vertigo** | **1** | **0** | **1** | **0** |
| **Hypertension** | **0** | **0** | **5** | **0** |
| **Polyneuropathy** | **3** | **0** | **2** | **0** |
| **Abdominal pain** | **2** | **0** | **2** | **0** |
| **Pain** | **5** | **0** | **4** | **0** |
| **Acute renal injury** | **0** | **0** | **2** | **1** |
| **Premature ventricular contractions** | **0** | **0** | **1** | **0** |
| **Deep vein thrombosis** | **1** | **0** | **0** | **0** |
| **Elevated liver enzymes** | **1** | **0** | **2** | **0** |
| **Myocardial infarction** | **0** | **0** | **2** | **2** |
| **Epistaxis** | **0** | **0** | **3** | **0** |

**Table S1**

**Adverse events**
