# Supplementary material for: Circulating Endothelial Progenitor Cells in Castration Resistant Prostate Cancer: A Randomized, Controlled, Biomarker Study
Source: PLoS One. 2014 Apr 22;9(4):e95310. doi: 10.1371/journal.pone.0095310 (PMC3995874; doi:10.1371/journal.pone.0095310)
Supplement: Protocol S1 — Study protocol as approved by the local ethics committee. (PDF) [file pone.0095310.s004.pdf]

RANDOMIZED, CONTROLLED BIOMARKER STUDY EVALUATING THE ANTI-ANGIOGENIC ACTIVITY OF SUNITINIB IN HORMONE REFRACTORY PROSTATE CANCER PATIENTS TREATED BY DOCETAXEL

**Study No**	**MK URO 04**
**EudraCT**	**2007-003705-27**
**Investigators:**	**Principal Investigator:**
	**Univ. Prof. Dr. Michael Krainer**
	**Senior Co-Investigator:**
	**Dr. Volker Wacheck**
**Sponsor:**	**Dept of Internal Medicine I,**<br>**Medical University Vienna, Austria**
**Protocol version:**	**Amended Version I1, 19.06.2008**

1 TABLE OF CONTENTS

1 TABLE OF CONTENTS 2
2 STUDY SYNOPSIS 4
3 BACKGROUND AND SCIENTIFIC RATIONALE 8
3.1 Prostate cancer and current therapy 8
3.2 Tumor vasculature and circulating endothelial cells 9
3.3 Sunitinib 10
3.4 Rationale 11
3.5 Hypothesis 12
4 OBJECTIVES 13
4.1 Primary objective 13
4.2 Secondary objectives 13
5 INVESTIGATIONAL PLAN 14
5.1 Study Design 14
5.2 Selection of Study Population 16
5.2.1 Number of patients 16
5.2.2 Inclusion criteria 16
5.2.3 Exclusion criteria 17
5.3 Concomitant Medication 17
5.4 Treatment duration 18
5.5 Discussion of study design: 18
5.1 Trial duration and termination 19
6 OUTCOME MEASURES 20
6.1 Primary Outcome Measure 20
6.2 Secondary Outcome Measures 20
7 STUDY PROCEDURES 21
7.1 Screening Procedures 21
7.2 Randomization Procedure 22
7.3 Treatment Period Procedures 23
7.3.1 Phase 1: 23
7.3.2 Phase 2: 24
7.4 End of Study visit 25
8 STUDY MEDICATION 26
8.1 Sunitinib 26
8.1.1 Pharmcokinetics 27

8.1.2 Undesirable effects 27
8.1.3 Combination with docetaxel: 27
8.2 Standard of care docetaxel 28
8.2.1 Pharmacokinetics 29
8.2.2 Docetaxel premedication 30
9 DOSE MODIFICATIONS 30
9.1 Docetaxel mandatory dose modifications and treatment alterations 30
9.2 Sunitinib mandatory dose modifications and treatment alterations 34
10 ADVERSE EVENTS (AES) AND SERIOUS ADVERSE EVENTS (SAES) 36
10.1 DEFINITION OF ADVERSE EVENT, ADVERSE DRUG REACTION AND SERIOUS adverse event 36
10.1.1 Adverse events (or adverse experience) (AE): 36
10.1.2 Adverse drug reaction (ADR): 36
10.1.3 Serious adverse event or reaction/experience (SAE): 37
10.2 Methods of recording and assessing adverse events 38
10.2.1 Procedure for reporting serious adverse events 39
10.3 Monitoring of patients with adverse events 40
11 STATISTICAL CONSIDERATIONS 40
11.1 Analysis of biomarker 40
11.2 Analysis of safety 40
11.3 Sample-size 41
12 ETHICAL AND REGULATORY ASPECTS 41
12.1 Risk/Benefit assessment 41
12.2 Responsibilities of the Investigator 41
12.3 Subject information and informed consent 42
12.4 Ethics Committee or Institutional Review Board 42
12.5 Notification to authorities 42
13 ADMINISTRATIVE CONSIDERATIONS 43
13.1 Case report form handling 43
13.2 Source data and subject files 43
13.3 Investigator site file and archiving 44
13.4 Monitoring, quality assurance and inspection by authorities 45
13.5 Changes to the study protocol 46
13.6 Study report and publication 46
14 REFERENCES 47
ATTACHMENT I 49
ATTACHMENT II 50
ATTACHMENT III 51

2 STUDY SYNOPSIS

**Study title**	Randomized, controlled biomarker study evaluating the anti-angiogenic activity of sunitinib in hormone refractory prostate cancer patients treated by Docetaxel
**Study number**	MK URO 4
**Sponsor**	Dept of Internal Medicine I, Medical University Vienna, Austria
**Study phase**	IIa
**Study location**	Dept of Internal Medicine I, Medical University Vienna, Austria
**Planned study period**	First patient in: Autumn 2007<br>Duration of recruitment: Winter 2008
**Summary**	Docetaxel (75mg/m^2 q21d) is standard of care for patients with hormone refractory prostate cancer (HRPC). Recent data indicate, that chemotherapeutics given at MTD induce, besides their cytotoxic effects, mobilization of circulating endothelial cells (CEC) and - progenitors (CEP) in drug-free breaks of each cycle. In preclinical models, mobilized CEC/CEP result in tumor vasculogenesis and progression of disease.<br>We hypothesize that treatment with sunitinib, an anti-angiogenic tyrosine kinase inhibitor, in between 3 weekly docetaxel disrupts CEC/CEP spikes following docetaxel leading to chemosensitization and reduced tumor re-growth in HRPC patients responding to docetaxel.
**Study objectives:**	<u>Primary:</u><br>- whether CEC/CEP spikes induced by MTD docetaxel are suppressed by sunitinib in patients treated with docetaxel/sunitinib relative to docetaxel monotherapy<br><br><u>Secondary</u>:<br>- whether docetaxel/sunitinib increase response rate and length of treatment holidays relative to docetaxel monotherapy<br>- CEC/CEP remain suppressed by sunitinib maintenance therapy relative to patients receiving no sunitinib during chemotherapy treatment holidays<br>- suppression of CEC/CEP by sunitinib co-treatment correlates with response rate and prolonged treatment holidays relative to patients receiving no sunitinib<br>- whether serum angiogenesis biomarkers (i.e. VEGF, VEGFR, TSP1) and tumor biomarker (i.e. Visfatin, SphP1) are suppressed by co-treatment with sunitinib and correlate with response rate and the prolongation of treatment holidays

	- whether treatment by sunitinib/docetaxel and sunitinib maintenance therapy is safe and tolerable
Study design and plan:	Exploratory, randomized controlled Phase IIa study with two consecutive treatment phases. Patients will be randomized at a 1:1 ratio to one of two treatment arms in treatment Phase 1:<br>**Phase 1:**<br>- ***Arm A***: - docetaxel 75mg/m^2 day1 q 21d x 4 cycles,<br>- prednisolone 2 x 5 mg qd for cycle III-IV<br>- sunitinib 37.5mg/d day2 –day15 x 4 cycles<br><br>- ***Arm B***: - docetaxel 75mg/m2 day1 q 21d x 4 cycles,<br>- prednisolone 2 x 5 mg qd for cycle III-IV<br><br>After four cycles patients will be assessed by PSA and radiologic examination for response. Only patients responding to treatment given in Phase 1 as defined by a PSA decline of ≥ 50% compared to baseline and no objective progression according to modRECIST are eligible for Phase 2 of the trial. All other patients (i.e. non-responders) discontinue treatment and will be excluded from the study.<br><br>**Phase 2:**<br>Responders to docetaxel + sunitinib in Phase 1 will be randomized in Phase 2 to receive either<br>***Arm A***: sunitinib maintenance therapy (50mg/d for 14 days followed by one week rest) or<br>***Arm B***: no treatment during their chemotherapy holidays.<br>Responders to docetaxel monotherapy *in* Phase 1 will receive no treatment during their chemotherapy holidays (***Arm C***).<br>During chemotherapy holidays PSA-levels will be measured every three weeks. Every two months radiologic examinations will be performed (only pts with measurable disease).<br>Treatment with sunitinib or no treatment, respectively, will be prolonged as long as the patients have no PSA-progression above baseline. Whenever a patient progresses with a PSA above his baseline value or a new radiologic lesion or a symptomatic progress develops, his participation in the study will be terminated.
**Planned number of subjects**	60 HRPC patients
**Diagnosis and main criteria for inclusion and exclusion**	**<u>Inclusion criteria:</u>**<br>• Signed written informed consent<br>• male patients ≥ 18 years of age

- WHO performance status of 0-2.
- Histologically proven prostate adenocarcinoma.
- All patients must have prostate adenocarcinoma that is unresponsive or refractory to androgen ablation with biochemical progression
- Measurable and/or evaluable progressive disease, which is defined by one of the following three criteria:
  - 25% increase in bidimensionally measurable soft tissue metastases
  - Appearance of new metastatic lesions (proven by CT scan, X-ray or bone scan)
  - PSA level of at least 10ng/mL, with increases on at least 2 successive occasions at least 2 weeks apart
- If the patient has been treated with antiandrogens, treatment must have been stopped at least 6 weeks prior to study randomization
- Continuation of biphosphonates is allowed
- Baseline LVEF 50 % measured by echocardiography or MUGA scan
- Adequate bone marrow reserve:
- WBC ≥ 3.5 x 10^9/L, ANC ≥ 1.5 x 10^9/L, Hb ≥ 10 g/dl, platelets ≥ 100 x 10^9/L
- Adequate liver function:
- Bilirubin ≤ 1, 5 x UNL (upper normal level), ASAT and ALAT ≤ 1,5 x UNL
- Adequate renal function:
- Creatinine ≤ 1,5 times the UNL

Exclusion Criteria:

- Prior chemotherapy, radiotherapy, involving more than 25% of bone marrow producing area
- Known brain metastases
- Peripheral neuropathy > grade 1 (according to NCI-CTC version 3.0)
- Prior malignancy except the following: adequately treated basal cell or squamous cell skin cancer, or any other cancer from which the patient has been disease-free for ≥5 years
- Active infection or known HIV
- Other serious illness or medical condition: Concurrent treatment with other experimental drugs. Participation in another clinical trial with any investigational drug within 30 days prior to study screening
- Presence of any psychological, familial, sociological, geographical

	condition hampering compliance with the study protocol and follow up schedule
Investigational product: Dose/mode of administration/dosage schedule	Sunitinib will be administered orally in form of tablets (Phase 1:37.5mg/d; Phase 2: 50mg/d) on a three-week repeating schedule, with two weeks of daily treatment followed by a one-week rest.
Risk /benefit assessment	No standard treatment or concomitant medication will be withheld from any patient. All HRPC patients will receive first choice standard chemotherapy with docetaxel. Sunitinib, which has already been approved for metastatic renal cell cancer and GIST and was tolerated in a Phase I in combination with docetaxel, will be administered orally as an anti-angiogenic drug for improving tumor response to docetaxel. Anticipated and particularly closely monitored side effects include primarily haematological parameters. The potential benefits appear to clearly outweigh the risks.

3 BACKGROUND AND SCIENTIFIC RATIONALE

3.1 Prostate cancer and current therapy

Prostate cancer is the most frequently diagnosed male cancer and accounts for the second most common reason for cancer specific death in men (1). For patients with hormone refractory advanced stage of the disease, systemic chemotherapy is the only available treatment option. Docetaxel has shown clinical significant activity in two independent clinical phase III trials recently and is therefore considered standard of care in this patient population.

Docetaxel is a semisynthetic taxane derived from a precursor extracted from the needles of the European yew, Taxus baccata. It has a mechanism of action which is similar to (or may be identical to) paclitaxel. Docetaxel enhances microtubule assembly and inhibits the depolymerization of tubulin. As with paclitaxel, this can lead to bundles of microtubules in the cell, which by blocking cells in the M phase of the cell cycle results in the inability of the cells to divide. This contrasts with the action of other spindle poisons in clinical use such as colchicine or vinca-alkaloids which inhibit tubulin assembly in microtubules.

Recent data from two randomized studies, TAX327(2), and SWOG 9916(3), which compared docetaxel-based chemotherapy to mitoxantrone-based therapy, have demonstrated that treatment with docetaxel can prolong life in a statistically significant way in patients with HRPC. In the TAX237 trial the median overall survival rates for patients treated with docetaxel every 3 wk was 18.9 mo, compared with 16.4 mo for the patients in the control arm (p=0.009). Patients treated with the combination of docetaxel and estramustine in the SWOG trial had a significant improvement in median survival (18 mo vs 16 mo, p=0.01), longer progression-free survival (6 mo compared with 3 mo, p<0.0001), and a 20% reduction in the risk of death. These results have altered the general perception about response to chemotherapy in HRPC and docetaxel has become the standard of care for this patient group.

While docetaxel/prednisone chemotherapy has demonstrated a survival advantage in metastatic HRPC patients, the optimal duration of chemotherapy has not yet been established. Currently, a standard practice is to treat patients indefinitely until unacceptable toxicity or disease progression. A systematic approach to providing breaks in treatment schedules (intermittent chemotherapy) for patients who experience an initial response to chemotherapy may avoid or delay the development of progressive toxicity. A recent clinical trial studied for the first time intermittent docetaxel treatment (Beer, ASCO abstract, 2006). After four cycles

docetaxel treatment was suspended in patients responding to docetaxel. Whenever these patients progressed during treatment holiday (median 17 weeks), treatment by docetaxel was re-started. Re-treatment by docetaxel resulted in major PSA response or PSA stabilisation in 50% and 35% of these patients, respectively.

Despite these encouraging data there is still a clear need for more effective treatment of HRPC both in terms of initial response to docetaxel and the duration of treatment holidays in patients responding to docetaxel.

3.2 Tumor vasculature and circulating endothelial cells

The tumor vasculature has emerged as a clinically validated therapeutic target (4, 5). Gaining access to the hosts vascular system and the maintenance of sufficient blood supply are growth-limiting steps in tumour progression. Most tumours form endothelial cell based vessels by angiogenesis, the sprouting of new vessels from existing vessels, but also an adapted form of the embryonic process of vasculogenesis can be observed, where blood vessels are formed de novo by circulating endothelial progenitor cells (CEP) (6). CEPs are a subpopulation of circulating endothelial cells with a progenitor-like phenotype. They can be mobilised from the bone marrow, transported through the blood stream to become incorporated into the walls of growing blood vessels. CEPs can be mobilised from the bone marrow and recruited to the sites of neoangiogenesis by angiogenic growth factors as VEGF, angiopoietin and erythropoietin (reviewed in (7, 8)). Preclinical and clinical data indicate that these CEPs can incorporate in cancer vessels, albeit usually at low frequencies. Some preclinical studies suggest that CEPs have a key role in promoting cancer vasculogenesis and in late stages of cancer development (4).

In the circulation of adults, more mature CEPs are found that have lost CD133, but are still positive for VEGFR-2 and CD34. These circulating endothelial cells (CEC) with a mature phenotype, which are probably derived from blood vessel wall turnover, are increased in patients with some types of cancer and in various other conditions including mechanical, inflammatory, infective, ischaemic and autoimmune states. These CEC are mostly viable and exhibit still proliferative capacity despite their terminal differentiation(8).

In summary, there is growing evidence that CEP/CEC can contribute to tumour angiogenesis, but such contributions can vary depending on circumstances such as tumour grade, tumour stage, location of tumour growth and whether anticancer therapy has recently been initiated (4).

3.3 Sunitinib

Sunitinib is a small molecule inhibiting multiple receptor tyrosine kinases (RTKs) associated with tumor growth, pathological angiogenesis and metastatic progression of tumor disease. On a molecular level sunitinib shows targeted blockade of the signal pathways of growth factors by inhibition of the following associated tyrosine kinase receptors:

- vascular endothelial growth factor receptor (VEGFR1, VEGFR2, VEGFR3)
- platelet-derived growth factor receptor (PDGFR-a, PDGFR-ß)
- KIT (stem cell factor) receptor
- FLT-3 (Fms-like tyrosine kinase-3) receptor
- RET (rearranged during transfection) receptor
- CSF1 (colony stimulating factor 1) receptor

Sunitinib shows both antiangiogenic and antitumor activity by blocking receptors promoting angiogenesis as such (VEGRF and PDGFR) and simultaneously inhibiting receptors stimulating tumor growth (PDGFR, KIT, or RET) which will result in a disruption of tumor cell production while inducing the death of tumor cells. The primary active metabolite shows a similar potency as sunitinib.

Sunitinib (SUTENT) is currently approved for the treatment of advanced and/or metastatic renal cell carcinoma (MRCC) and for the treatment of unresectable and/or metastatic malignant gastrointestinal stromal tumors (GIST) after failure of treatment with imatinib mesylate due to resistance or intolerance.

For prostate cancer sunitinib has been already studied in combination with safety and anti-tumor activity in preclinical and clinical settings. Recent preclinical experiments have demonstrated sunitinib single agent activity as well as supra-additive anti-tumor activity with docetaxel in a prostate cancer xenograft model (9).

The dose of docetaxel in combination with sunitinib to be used in the investigational arm of this study has been selected based on the results of a Phase 1 study conducted in the US. The maximum tolerated dose (MTD) of docetaxel at 75 mg/m2 was combined with sunitinib given at 37.5 mg/day (Schedule 2/1). Preliminary data of non- hematological and hematological adverse events are consistent with the observations seen in patients who

receive docetaxel monotherapy. The most frequently observed adverse events were neutropenia, fatigue and taste alteration. In this study, the schedule 2/1 for sunitinib has been selected in order to have a 1-week wash-out period for sunitinib and its primary active metabolite SU012662 before docetaxel infusion. Since both sunitinib and docetaxel are metabolized hepatically by the cytochrome P450 enzyme system (CYP3A4), the 1-week wash-out period before docetaxel should minimize any possible pharmacokinetic interaction of sunitinib at steady-state with docetaxel.

3.4 Rationale

Antiangiogenic therapy is a promising new form of cancer treatment and the effectiveness of new angiogenesis inhibitors (e.g. sunitinib) are currently tested in several studies.

An antiangiogenic effect may be reflected by a decrease of CEP/CEC. In previous studies, mature CEC and progenitor cells in peripheral blood of breast cancer patients were modulated in response to chemotherapy (including taxanes) and correlated with serum levels of angiogenic growth factors as well as classic clinical and pathological features of breast cancer disease (6, 10).

Results of recent clinical trials as well as preclinical data indicate that treatment by anti-angiogenic therapeutics (e.g., sunitinib) might be a promising novel strategy to chemosensitize human tumors to standard therapy. Also for standard chemotherapy regimen as taxanes and cyclophosphamide antiangiogenic properties are discussed if administered at maximum-tolerated dose (MTD) (11, 12).

MTD chemotherapy is able to cause like vascular disrupting agents (VDA) acute occlusion of existing blood vessels leading to a rapid and massive intratumoral necrosis (13). However, such tumors rapidly regrow due to an acute mobilization of bone marrow–derived circulating endothelial progenitor cells, which home to the viable tumor rim that characteristically remains after such therapy (13). Disruption of this CEP spike by anti-angiogenic drugs resulted in marked reductions in tumor rim size and blood flow as well as enhanced VDA antitumor activity in vivo. CEPs are induced to leave the bone marrow and enter the vasculature by circulating angiogenic factors such as VEGF.

Only recently, Guerin et al. demonstrated supra-additive anti-tumor effect sunitinib-docetaxel combination in a human prostate xenograft mouse model (9). For humans it has been shown, that CEP are mobilised by taxane based chemotherapy in breast cancer patients (6). Since this

mobilisation of CEP may contribute to tumour neovascularisation it has been suggested that concomitant treatment by VEGF targeting anti-angiogenic drugs (like sunitinib) impedes tumor neo-angiogenesis induced by standard chemotherapeutics administered at maximum tolerated doses.

3.5 Hypothesis

We hypothesize that treatment with sunitinib disrupts CEP/CEC spikes following treatment by docetaxel leading to chemosensitization and reduced tumor re-growth in HRPC patients responding to docetaxel.

4 OBJECTIVES

4.1 Primary objective

The primary objective of the study is to evaluate

- whether CEC/CEP spikes induced by MTD docetaxel are suppressed by sunitinib in patients treated with docetaxel/sunitinib relative to docetaxel monotherapy

4.2 Secondary objectives

The secondary objectives are to assess whether

- docetaxel/sunitinib increase response rate and length of treatment holidays relative to docetaxel monotherapy
- CEC/CEP remain suppressed by sunitinib maintenance therapy relative to patients receiving no sunitinib during chemotherapy treatment holidays
- suppression of CEC/CEP by sunitinib co-treatment correlates with response rate and prolonged treatment holidays relative to patients receiving no sunitinib
- whether serum angiogenesis biomarkers (i.e. VEGF, VEGFR, TSP1) and tumor biomarkers (i.e. Visfatin, SphP1) are suppressed by co- treatment with sunitinib and correlate with response rate and treatment holidays
- whether treatment by sunitinib/docetaxel and sunitinib maintenance therapy is safe and tolerable

5 INVESTIGATIONAL PLAN

5.1 Study Design

This is an exploratory, randomized controlled, open-label Phase IIa study in an "add-on" design with two consecutive treatment phases.

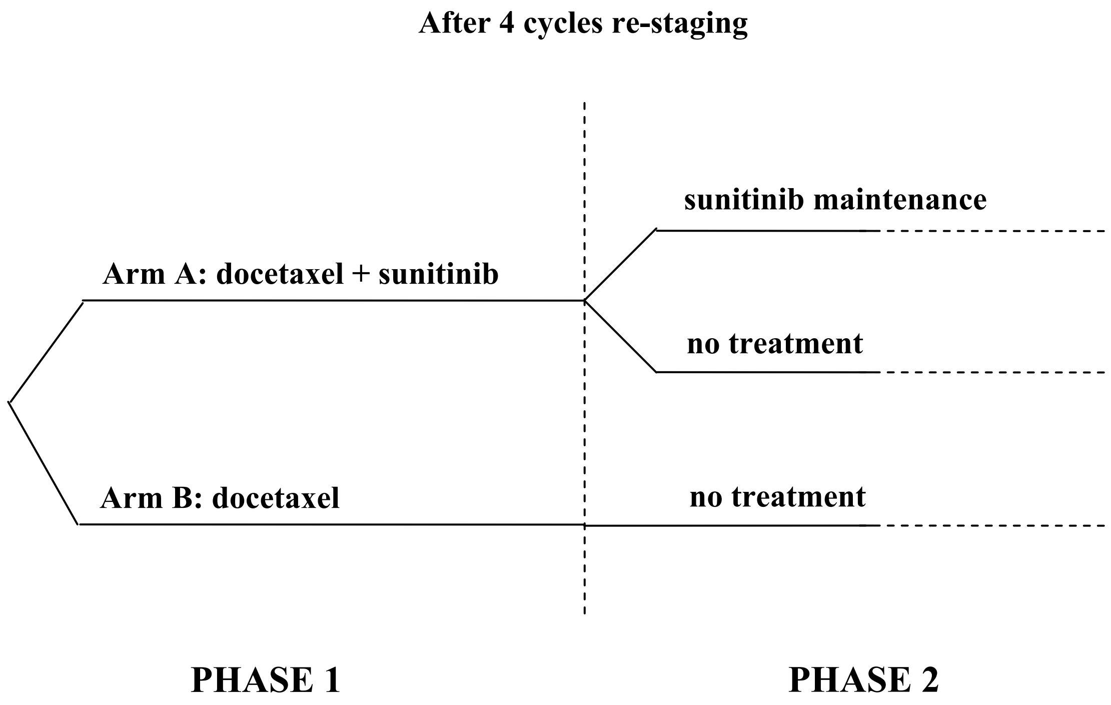

Phase 1:

A total of 60 patients with HRPC will be randomized in a 1:1 ratio to both treatment arms of the first treatment phase.

In Phase 1 of the study, all patients will receive standard of care therapy by docetaxel 75mg/m^2 q 21d x 4. Prednisolone 2 x 5 mg per day continuously will be administered beginning from cycle III.

In addition to standard therapy patients randomized in *Arm A* will receive sunitinib (37.5mg/d) on a three-week repeating schedule, with two weeks of daily treatment followed

by a one-week rest starting the day after each docetaxel administration.

After four cycles of docetaxel treatment patients in both arms will be assessed by PSA and radiologic examination (only pts with measurable disease) for response.

Phase 2:

Only patients responding to treatment given in Phase 1 as defined by a PSA decline of ≥ 50% compared to baseline and no objective progression according to modRECIST are eligible for Phase 2 of the trial. All other patients (i.e. non-responders) discontinue and will be excluded from the study.

Responders to docetaxel + sunitinib in Phase 1 will be randomized in Phase 2 to receive either sunitinib maintenance therapy (50mg/d for 14 days followed by one week rest, *Arm A*) or no treatment during their chemotherapy holidays (*Arm B*).

Responders to docetaxel treatment in Phase 1 will receive no treatment during their chemotherapy holidays (*Arm C*).

During chemotherapy holidays PSA-levels will be measured every three weeks. Every two months radiologic examinations will be performed (only pts with measurable disease).

Treatment with sunitinib or no treatment, respectively, will be prolonged as long as the patients have no PSA-progression above baseline. Whenever a patient progresses with a PSA above his baseline value or a new radiologic lesion or a symptomatic progress develops, his participation in this study will be terminated.

5.2 Selection of Study Population

5.2.1 Number of patients

A total number of 60 patients will be enrolled in this trial corresponding to 30 pts for each arm in Phase 1

5.2.2 Inclusion criteria

All patients entering the study must comply with all of the following criteria:

- Signed informed consent prior to beginning protocol specific procedures.
- Male subjects with a minimum age of 18 years.
- WHO performance status of 0-2.
- Histologically proven prostate adenocarcinoma.
- All patients must have prostate adenocarcinoma that is unresponsive or refractory to androgen ablation with biochemical progression.
- Measurable and/or evaluable progressive disease, which is defined as one of the following three criteria:
  - 25% increase in bidimensionally measurable soft tissue metastases.
  - Appearance of new metastatic lesions (proven by CT scan, X-ray or bone scan).
  - PSA level at least 10 ng/mL, with increases on at least 2 successive occasions at least 2 weeks apart.
- Castrate level of testosterone (<50ng/dl) in all patients. Patients with medical castration with LH-RH analogue must continue analogue.
- If the patient has been treated with antiandrogens, treatment must have been stopped at least 6 weeks prior to study randomization.
- Baseline LVEF 50 % measured by echocardiography or MUGA scan
- Continuation of biphosphonates allowed.

- Adequate bone marrow reserve (WBC ≥ 3.5 x 10^9/L, ANC ≥ 1.5 x 10^9/L, Hb ≥ 10 g/d^l, platelets ≥ 100 ^{x} 10^9/L).

- Adequate liver function: (Bilirubin ≤ 1, 5 x UNL (upper normal level), ASAT and ALAT ≤ 1,5 x UNL).

- Adequate renal function (creatinine ≤ 1,5 times the UNL)

5.2.3 Exclusion criteria

No patient may be included into the study who fulfills **any** of the following criteria

- Known brain metastases.
- Peripheral neuropathy > grade 1 (according to NCI-CTC version 3.0).
- Prior malignancy except the following: adequately treated basal cell or squamous cell skin cancer, or any other cancer from which the patient has been disease-free for $\geq$5 years.
- Increasing analgesic use.
- Prior chemotherapy, radiotherapy, involving more than 25% of bone marrow producing area. (Prior use of Estramustine phosphate is allowed).
- Active infection or known HIV.
- Other serious illness or medical condition: unstable cardiac disease despite treatment, myocardial infarction within 6 months prior to study entry, active uncontrolled infection, peptic ulcer, unstable diabetes mellitus or other contraindications for the use of prophylactic corticosteroid medication.
- Concurrent treatment with other experimental drugs. Participation in another clinical trial with any investigational drug within 30 days prior to study screening.
- Presence of any psychological, familial, sociological, geographical condition hampering compliance with the study protocol and follow up schedule.

5.3 Concomitant Medication

Palliative and supportive care for disease and treatment-related symptoms and syndromes will be offered to all patients in this trial.

The following treatments are not permitted during the treatment phase:

- Other anticancer drugs
- Investigational drugs

5.4 Treatment duration

Treatment with sunitinib or no treatment, respectively, will be prolonged as long as the patients have no PSA-progression above baseline. Whenever a patient progress with a PSA above his baseline value or a new radiologic lesion or a symptomatic progress develops, his participation in this study will be terminated.

Furthermore, patients with occurrence of one of the above events, patients will be discontinued.

- symptomatic deterioration
- unacceptable toxicity to docetaxel and/or sunitinib
- the trial treatment is repeatedly not administered as specified by the protocol
- withdrawal of informed consent
- withdrawal by the managing physician if he feels it is in the best interest of the patient

5.5 Discussion of study design:

This is an exploratory study with the primary objective to investigate the anti-angiogenic activity of sunitinib as an add-on therapy to docetaxel for prostate cancer. By this, the study results will provide valuable insights into the mechanism of action of sunitinib in combination with a chemotherapeutic and anti-angiogenic biomarkers in patients (Phase 1). Furthermore, the study will evaluate sunitinib as a novel treatment strategy for maintenance therapy for patients initially responding to docetaxel + sunitinib (Phase 2).

In order to judge adequately the effect of sunitinib on the PD biomarkers all control groups outlined for Phase 1 and 2 are mandatory. CEP/CEC and VEGF/TSP1 levels will be evaluated as a pharmacodynamic (PD) biomarker for sunitinib`s anti-angiogenic activity and correlated to the objective response rate (Phase 1) and the length of treatment holidays (Phase 2).

Prednisolone co-treatment will started beginning from cycle III in all arms of Phase I for studying its effect on biomarkers relative to the first two cycles.

The Phase 2 of the study clearly extends the relevance of this project by generating PD data for persistence of anti-angiogenic activity of sunitinib from Phase 1 in the "no treatment" group as well as providing potentially "proof of concept" data for a role of sunitinib as a novel rational maintenance therapy. This maintenance therapy part is of particular interest since there is currently a lack of well defined treatment guidelines for patients initially responding to docetaxel. Treatment of patients randomized in Phase 1 to Arm B will be considered as standard of care with intermittent docetaxel treatment (Beer, ASCO abstract, 2006) and facilitates appreciation of external validity of results relative to other studies.

A total number of 60 patients will be enrolled in this trial corresponding to 30 pts for each arm in Phase 1. According to the study of Tannock et al (2), we expect about 50% of patients to respond to docetaxel single therapy. This sample size is considered sufficient for PD-analysis of the primary objective. There is no statistical justification for the sample size. PD parameters will be summarized by descriptive statistics.

In summary, besides the implications for future trials studying the clinical impact of sunitinib and docetaxel for prostate cancer treatment, the definition of a PD biomarker predicting sunitinib anti-angiogenic activity would provide a valuable tool for dose finding studies and for selecting study populations profiting most by a sunitinib co-treatment.

5.1 Trial duration and termination

It is anticipated that the screening of patients will start in autumn 2007 and will stop after the inclusion of 60 patients, which is expected in 2 years after initiation of the study.

6 OUTCOME MEASURES

6.1 Primary Outcome Measure

The primary objective of CEC/CEP spikes suppression by sunitinib in patients treated with docetaxel/sunitinib will be measured by FACS. Quantification of CEC/CEP from peripheral blood (5ml) will be performed according a protocol following the guidelines provided by the Bertolini/Kerbel group (4, 10).

CEC/CEP kinetics will be determined at each cycle of study Phase 1:

- Baseline,
- day 1 (i.e. before docetaxel administration),
- day 3 (i.e. ~24 h after sunitinib)
- day 8
- day 15 (i.e. end of sunitinib).

6.2 Secondary Outcome Measures

For assessment of the secondary objectives the following outcome measures will be performed:

- <u>docetaxel/sunitinib increase response rate and length of treatment holidays</u>
  tumor response at the end of study Phase 1 and time to progression in Phase 2 (= length of treatment holidays) will be determined by PSA measurements and CT-MRT scans.
- <u>CEC/CEP remain suppressed by sunitinib maintenance therapy during chemotherapy holidays</u>
  CEC/CEP of patients eligible for Phase 2 will be quantified as outlined above. CEC/CEP kinetics will be determined each cycle of study Phase 2
  - day 1 (i.e. before initiation of sunitinib (Arm A))
- <u>suppression of CEC/CEP by sunitinib co-treatment correlates with response rate and prolonged treatment holidays:</u>

CEC/CEP kinetics (AUC) will be quantified as outlined above and correlated with tumor response at the end of study Phase 1 and length of treatment holidays.

- <u>suppression of serum angiogenesis biomarkers (i.e. VEGF, VEGFR, TSP1) and tumor biomarker (i.e. Visfatin, SphP1) by sunitinib correlate with response rate and treatment holidays</u>

  VEGF, VEGFR, TSP1 and Visfatin will be quantified from peripheral blood (5ml) by commercially available ELISA or HPLC (SphP1). Biomarker kinetics will be determined each cycle
  - Phase 1 (day 1, day 3, day 8 and day 15)
  - Phase 2 (day 1 and day 15)
- <u>safety and tolerability of sunitinib and docetaxel</u>

  Safety and tolerability will be assessed be assessed by physical examinations and lab tests at the time point as indicated. Lab tests will comprise check of
  - Renal function: Serum creatinine
  - Liver function tests: Bilirubin, alkaline phosphatase, ASAT, ALAT, total protein
  - Hematological function: Hemoglobin, WBC, neutrophils, thrombocytes
  - Chemistry: Sodium, potassium
  - Cardiovascular function: Blood pressure, ECG

7 STUDY PROCEDURES

Patients with HRPC will be invited for participation in this trial. Informed consent must be obtained prior to any trial-specific procedures.

7.1 Screening Procedures

The following investigations must be performed within 28 days before start of study:

- Abdominal and thoracic CT scan or MRI. The same method of assessment should be used for all subsequent assessments
- In case the CT/MRI scan of the chest shows no metastatic lesions at baseline it is

allowed to follow this anatomy with a chest X-ray in the next visits. However, in that case a baseline chest X-ray must be available. In case of suspicion of lung metastases a CT/MRI scan should be performed for confirmation.

- Bone scan
- Echocardiography or MUGA scan
- Blood sampling for PSA and testosterone

The following investigations must be performed within 14 days before start of study:

- Medical history
- Check of inclusion/exclusion criteria
- Documentation of concomitant medication
- Vital signs: body temperature, heart rate and blood pressure (systolic and diastolic) after 5 minutes supine
- Physical exam including WHO performance status
- Weight and Height
- Blood sampling for clinical chemistry (alkaline phosphatase, ASAT, ALAT, bilirubin, creatinine, LDH, total protein, sodium, potassium, amylase, lipase)
- Blood sampling for hematology (hemoglobin, WBC, neutrophils, thrombocytes)
- Blood sampling for PD measurement
- ECG

7.2 Randomization Procedure

The investigator must review the results of each patient screening evaluation to assure that all eligibility criteria have been satisfied. The results of the assessment are documented in the patients Case Report Form.

If the patient is eligible for the study he will be enrolled into the study and a Subject Identification Number consisting of 3 digits will be assigned.

Patients will be assigned to treatment groups according to a randomization list to arm A or B in Phase 1.

7.3 Treatment Period Procedures

7.3.1 Phase 1:

Patients eligible for the study will be randomized to treatment arm A or B in Phase 1. The following procedures apply for patients in both treatment arms.

7.3.1.1 Day 1 of each cycle

The following investigations and procedures will be performed every day 1of each cycle before administration of study medication:

- Blood sampling for clinical chemistry
- Blood sampling for hematology
- Blood sampling for PSA
- Blood sampling for PD measurement
- ECG
- Vital signs
- Physical exam, including WHO performance status
- Recording of adverse events/concomitant medication/premature discontinuation

Following these procedures standard of care treatment by docetaxel 75mg/m^2 will be administered.

7.3.1.2 Day 3, 8 and 15 of each cycle:

The following investigations and procedures will be performed:

- Blood sampling for PD measurements
- Blood sampling for hematology
- Blood sampling for hematology and clinical chemistry (only day 8)

7.3.1.3 Tumor response evaluation visits after cycle 4:

The following investigations and procedures will be performed:

- CT scans or MRI of abdomen and thorax (if applicable) will be performed after 4 cycles for objective response evaluation. Response is to be defined according to modified *Response Evaluation Criteria in Solid Tumors (RECIST).* Tumor response does not need to be confirmed.
- Chest X-ray: if baseline CT/MRI assessment reveals no lung metastases, chest-X-ray should be repeated after cycle 4. In case of suspicion of lung metastases a CT/MRI scan should be performed to confirm this.
- Bone scan (if applicable)
- Blood sampling for PSA
- Blood sampling for PD measurement
- Physical examination, including assessment of WHO performance status

Only patients responding to treatment given in Phase 1 as defined by a PSA decline of ≥ 50% compared to baseline and no objective progression according to modRECIST are eligible for Phase 2 of the trial. All other patients (i.e. non-responders) discontinue and will be excluded from the study.

7.3.2 Phase 2:

Responders to docetaxel + sunitinib in Arm A will be randomized in Phase 2 to receive either sunitinib maintenance therapy (50mg/d for 14 days followed by one week rest) or no treatment during their chemotherapy holidays.

Responders to docetaxel treatment in Arm B will receive no treatment during their chemotherapy holidays.

7.3.2.1 Day 1 of each cycle

The following investigations and procedures will be performed every day 1of each cycle before administration of study medication:

- Blood sampling for clinical chemistry
- Blood sampling for hematology

- Blood sampling for PSA
- Blood sampling for PD measurement
- ECG
- Vital signs
- Physical exam, including WHO performance status
- Recording of adverse events/concomitant medication/premature discontinuation

Following these procedures sunitinib treatment 50mg/d will be initiated for 14 days according to randomization to treatment arms.

7.3.2.2 Day 15 of each cycle:

The following investigations and procedures will be performed:

- Blood sampling for hematology and clinical chemistry
- Blood sampling for PD measurement

7.3.2.3 Radiologic examinations every 4 cycles:

Every four cycles (i.e. three months) radiologic examinations will be performed (only pts with measurable disease).

- CT scans or MRI of abdomen and thorax (if applicable) will be performed after 4 cycles for objective response evaluation. Response is to be defined according to modRECIST criteria.
- Chest X-ray: if baseline CT/MRI assessment reveals no lung metastases, chest-X-ray should be repeated after cycle 4. In case of suspicion of lung metastases a CT/MRI scan should be performed to confirm this.
- Bone scan (if applicable).

7.4 End of Study visit

The End of Study Visit must be performed to follow up on the adverse events which were still ongoing during the last on study visit. The End of Study visit must be conducted no earlier than 30 days after the last administration of study medication. The following will be

performed and documented:

- Physical examination, including assessment of WHO performance status and check of vital signs
- Blood sampling for clinical chemistry
- Blood sampling for hematology
- Blood sampling for PSA
- Blood sampling for PD measurement
- Outcome of AEs ongoing at the last on study Visit
- Concomitant medication
- ECG
- Echocardiography or MUGA scan

A "Report of patient death" form must be completed when patients die. An "End of Study visit" form also has to be completed for all patients who die, and when the study terminates in patients who withdraw consent.

8 STUDY MEDICATION

8.1 Sunitinib

Sunitinib (Sutent®) is a small molecule inhibiting multiple receptor tyrosine kinases (RTKs) associated with tumor growth, pathological angiogenesis and metastatic progression of tumor disease. On a molecular level sunitinib shows targeted blockade of the signal pathways of growth factors by inhibition of the following associated tyrosine kinase receptors:

- vascular endothelial growth factor receptor (VEGFR1, VEGFR2, VEGFR3)
- platelet-derived growth factor receptor (PDGFR-□, PDGFR-ß)
- KIT (stem cell factor) receptor
- FLT-3 (Fms-like tyrosine kinase-3) receptor
- RET (rearranged during transfection) receptor
- CSF1 (colony stimulating factor 1) receptor

Sunitinib shows both antiangiogenic and antitumor activity by blocking receptors promoting angiogenesis as such (VEGRF and PDGFR) and simultaneously inhibiting receptors stimulating tumor growth (PDGFR, KIT, or RET) which will result in a disruption of tumor cell production while inducing the death of tumor cells.
SUTENT is currently approved for the treatment of advanced and/or metastatic renal cell carcinoma (MRCC) and for the treatment of unresectable and/or metastatic malignant gastrointestinal stromal tumors (GIST) after failure of treatment with imatinib mesylate due to resistance or intolerance.

8.1.1 Pharmcokinetics

Maximum concentrations (Cmax) of sunitinib are generally observed at times of 6 to 12 hours post oral dosing. Food has no effect on the bioavailability of sunitinib. Binding of sunitinib and its active metabolite to plasma proteins in in vitro assays was 95% and 90%, respectively. Sunitinib is primarily metabolized by the CYP3A4 enzyme. Elimination half-life of sunitinib and its primary active metabolite is about 40 to 60 hours and 80 to 110 hours, respectively. The primary active metabolite shows a similar potency as sunitinib.

8.1.2 Undesirable effects

The most important treatment-related serious adverse events associated with SUTENT treatment of patients with solid tumours were pulmonary embolism (1%), thrombocytopenia (1%), tumour haemorrhage (0.9%), febrile neutropoenia (0.4%), and hypertension (0.4%). The most common treatment-related adverse events (experienced by at least 20% of the patients) of any grade included: fatigue; gastrointestinal disorders, such as diarrhoea, nausea, stomatitis, dyspepsia and vomiting; skin discolouration; dysgeusia and anorexia. Fatigue, hypertension and neutropoenia were the most common treatment-related adverse events of Grade 3 maximum severity and increased lipase was the most frequently occurring treatment-related adverse event of Grade 4 maximum severity in patients with solid tumours. Prolonged QT interval events occurred in < 0.1%

8.1.3 Combination with docetaxel:

The dose of docetaxel in combination with sunitinib to be used in the investigational arm of this study has been selected based on the results of a Phase 1 study conducted in the US. The

maximum tolerated dose (MTD) was docetaxel at 75 mg/m2 combined with sunitinib given at 37.5 mg/day (Schedule 2/1). Preliminary data of non- hematological and hematological adverse events are consistent with the observations seen in patients who receive docetaxel monotherapy. The most frequently observed adverse events were neutropenia, fatigue and taste alteration. In this study, the schedule 2/1 for sunitinib has been selected in order to have a 1-week wash-out period for sunitinib and its primary active metabolite SU012662 before docetaxel infusion. Since both sunitinib and docetaxel are metabolized hepatically by the cytochrome P450 enzyme system (CYP3A4), the 1-week wash-out period before docetaxel should minimize any possible pharmacokinetic interaction of sunitinib at steady-state with docetaxel.

In the present study, in order to further minimize any interaction, sunitinib will be administered on Day 2, approximately 24 hours after docetaxel infusion, when docetaxel plasma levels are low.

Sunitinb will be provided as 12.5mg capsule by Pfizer.

8.2 Standard of care docetaxel

Docetaxel will be administered as standard of care for HRPC (2) at a dose of 75 mg/m2 administered over 1 hour for all patients enrolled in the study. Thus, docetaxel is by definition not an investigational study product.

Docetaxel is a semisynthetic taxane derived from a precursor extracted from the needles of the European yew, Taxus baccata. It has a mechanism of action which is similar to (or may be identical to) paclitaxel. Docetaxel enhances microtubule assembly and inhibits the depolymerization of tubulin. As with paclitaxel, this can lead to bundles of microtubules in the cell, which by blocking cells in the M phase of the cell cycle results in the inability of the cells to divide. This contrasts with the action of other spindle poisons in clinical use such as colchicine or vinca-alkaloids which inhibit tubulin assembly in microtubules.

Comparing paclitaxel and docetaxel using the "tubulin *in vitro* assay", the concentration required to provide 50% inhibition of microtubule disassembly (or IC50) for Docetaxel is 0.2 μm and for paclitaxel is 0.4 μm.

Complete available data on mechanism of action, experimental anti-tumor activity, preclinical data, safety and efficacy results from clinical trials in several tumor types and

pharmacokinetic profile of docetaxel can be found in the updated version of the Investigator Brochure.

8.2.1 Pharmacokinetics

Pharmacokinetic analysis performed in early phase I studies using 1-6 hours intravenous infusions every 3 weeks of docetaxel indicated that its elimination and distribution are linear *(14)*. Increases in AUC or in peak concentrations were observed with increases in docetaxel doses from 20 to 115 mg/m^2. Total plasma clearance was independent of dose. Docetaxel's pharmacological behaviour was best fitted by a three-compartment pharmacokinetic model with µ, b, and g half-lives of 4 minutes, 36 minutes, and 11.1 hours, respectively. However, when administered as a 24-hour continuous intravenous infusion or as a brief, daily infusion for 5 days, clearance was increased relative to short (1-6h) exposure schedules. A two- rather than a three compartment model provided a better fit of the plasma concentration data in the schedules achieving more prolonged exposure.

Following intravenous administration, docetaxel is heavily (98%) bound to plasma proteins. Elimination occurs principally by hepatic metabolism and biliary excretion. Up to 75-80% of radio-labelled docetaxel is recovered in the bile and feces, whereas less than 10% is excreted unchanged in the urine. Cytochrome P-450 enzymes are responsible for the metabolism of docetaxel, specifically the CYP-3A subfamily *(14)*.

Pharmacokinetic analysis of 24 phase II studies of 640 patients receiving docetaxel over 1 hour every 3 weeks, using four randomized limited-sampling schedules, confirmed the pharmacokinetic results of phase I trials and have permitted the evaluation of several prognostic factors as predictors of docetaxel-induced toxicity *(14)*. Clearance, AUC, and the duration of exposure over 0, 20 mmol/l (0,16 mg/ml) were all independent predictors for grade 4 neutropenia. Cumulative dose was the strongest predictor of the time to onset of fluid retention, however, measures of drug exposure were also covariates with independent predictive power.

Patients with concomitant elevations in hepatic enzymes (transaminases > 1,5 x ULN and alkaline phosphatase > 2,5 x ULN) in the above studies had a 27% reduction in docetaxel clearance, predicting for a 1,5-fold increase in the odds of febrile neutropenia. This finding motivated a specific safety analysis conducted in 1.366 patients from the entire docetaxel phase II clinical data base. Fifty-four patients (4%) who met the criteria for concomitant elevation in hepatic enzymes had a threefold higher incidence of febrile neutropenia during

course 1 than 1.312 patients with normal enzymes (22,6% vs. 6,2%, p<0,001) *(14)*. Several other safety parameters (severe infections, mucositis, and toxic death) were also markedly impaired in these patients. The results of pharmacokinetics studies in animals indicate that docetaxel exhibits multiphasic plasma kinetics, good tissue distribution, primarily metabolism and biliary excretion, and extensive excretion in the feces. After i.v. administration, docetaxel is distributed to all tissues and organs with the exception of the brain. It is highly bioavailable in tumor tissue. It crosses the blood-placental barrier and has been detected in maternal milk. It is eliminated very rapidly, although at a slower rate from tumor tissue than from normal tissue. Monoxygenase enzymes, in particular Cytochrome P-450-3A, play a leading role in docetaxel metabolism. Docetaxel binds strongly to plasma proteins in all species studied, including humans.

Docetaxel will be provided by the AKH hospital pharmacy as routine ready to use infusion bag. Patients will receive docetaxel intravenous infusions via infusion pump or gravity drip.

8.2.2 Docetaxel premedication

All patients will receive premedication with oral dexamethasone 8 mg twice daily for 3 days starting the day before docetaxel treatment in cycle I-II.

9 DOSE MODIFICATIONS

If necessary, treatment can be delayed for toxicity for a maximum of one week. In case of treatment delay both drugs have to be delay during combination therapy.

9.1 Docetaxel mandatory dose modifications and treatment alterations

Every effort will be made to administer the full dose regimen to maximize dose-intensity.

If possible, toxicities should be managed symptomatically. If toxicity occurs, the appropriate treatment will be used to ameliorate signs and symptoms including anti-emetics for nausea and vomiting, anti-diarrheals for diarrhea, and antipyretics and/or antihistamines for drug

fever.

If a patient experiences several toxicities and there are conflicting recommendations, the most conservative dose adjustment will be adopted.

No more than 2 dose reductions will be adopted per patient.

Doses should be adjusted according to the following recommendations (see table below).

Table: Dose levels for dose reduction

Dose Level	DOCETAXEL Q3 WEEKS
0	75 mg/m^2
-1	60 mg/m^2
-2	45 mg/m^2

Hematologic Toxicity:

Neutropenia and/or its complications:

Adverse event	Action to be taken
- Grade 4 neutropenia* for 7 days or more<br>- Grade 3-4 neutropenia* with oral fever $\geq$38.5°C<br>- Infection with grade 3-4 neutropenia*	If the patient develops one of these adverse events, the next infusion should be given with a one-level dose reduction.

Concomitant dose reduction of sunitinib will be considered only if sunitinib contribution to the event cannot be ruled out. [The median time to nadir of docetaxel is 7 days. Time to nadir of sunitinib may approach day 14-21 with schedule 2/1.]

Thrombocytopenia:

In case of grade ≥ 3 platelet-toxicity (NCI-CTC), delay maximum 2 weeks until platelets recover to ≥ 100 x 10^9/l, then treat with a one-level dose reduction.

Allergy (anaphylactic and hypersensitivity reactions) due to Docetaxel:

Hypersensitivity reactions that occur despite pre-medication are very likely to occur within a few minutes of start of the first or of the second infusion of docetaxel. Therefore, during the

1st and the 2nd infusion, careful evaluation of general sense of well-being and blood pressure and heart rate monitoring will be performed for at least the first 10 minutes, so that immediate intervention is possible in response to symptoms of an untoward reaction.

Facilities and equipment for resuscitation must be immediately available: antihistamine, corticosteroids, aminophylline, epinephrine.

If a reaction occurs, the specific treatment that is medically indicated for a given symptom (e.g. epinephrine in case of anaphylactic shock, aminophylline in case of bronchospasm, etc.) will be instituted.

Nausea/Vomiting:

A prophylactic anti-emetic treatment should be given to the patients from the first cycle. Anti-emetic prophylaxis with 5-HT3 Antagonists (i.e. ondansetron) should be given to all patients.

If despite even more aggressive antiemetic medication (i.e.aprepitant), grade ≥ 3 nausea/vomiting still occur, reduce the dose of study drug by one dose level. If despite dose reduction, nausea/vomiting still occur at grade ≥ 3, the patient will go off study.

Stomatitis:

Grade ≤ 2: no dose reduction, study chemotherapy should be withheld until resolution to grade ≤ 1.

If grade 3 stomatitis occurs, study drug should be withheld until resolution to grade ≤ 1. Treatment may then be resumed, but the dose of study drug should be reduced by one dose level for all subsequent doses.

In case of grade 4 stomatitis, the patient will go off study.

Peripheral neuropathy:

In case of signs or symptoms experienced by the patient, dose modification should be performed as follows:

Grade ≤ 1: no change.

Grade 2: re-treat with a one-level dose reduction (no further dose reduction is planned).

Grade ≥ 3: patient will go off protocol therapy.

Skin toxicity:

Grade ≤ 2: no change.

Grade 3: delay until ≤ grade 1, maximum two weeks then reduce dose of study drug by one dose level; if no recovery to ≤ grade 1 within two weeks, patient will go off protocol therapy.

Liver toxicity:

In case of increase of ASAT and/or ALAT to >1,5 x ULN or bilirubin to > ULN, delay study drug treatment for up to 2 weeks until ASAT and/or ALAT return to ≤ 1.5 x ULN and bilirubin to ≤ ULN. Then re-treat at one dose level lower. If no recovery to < 1,5 x ULN and bilirubin to < ULN within two weeks delay, patient will go off protocol therapy.

Docetaxel-induced fluid retention:

In case of fluid retention (peripheral edema and/or effusions) during treatment with docetaxel, the signs and symptoms should be graded as mild, moderate, severe or life-threatening as recommended in Appendix G.

NO DOSE REDUCTION IS PLANNED.

The patient's body weight will be recorded and followed as frequently as possible to document any weight gain which could be related to edema.

Docetaxel induced hyperlacrimation:

The excessive lacrimation (epiphora) seen in some patients receiving docetaxel appears to be related to cumulative dose (median ~300 mg/m^2) and resolves rapidly after treatment discontinuation.

Excessive lacrimation seems to be the result of a chemical conjunctivitis and/or chemical inflammation (with edema) of the lacrimal duct epithelium (producing a reversible lacrimal duct stenosis).

NO DOSE REDUCTION PLANNED.

In patients experiencing clinically significant hyperlacrimation, the following approach is recommended:

Frequent instillation of artificial tears. Prescribe a steroid ophthalmic solution (e.g. prednisolone acetate): 2 drops each eye bid for 3 days starting the day before docetaxel administration in patients without history of herpetic eye disease.

Alopecia and nail changes:

NO DOSE REDUCTION PLANNED.

9.2 Sunitinib mandatory dose modifications and treatment alterations

Sunitinib will be administered orally for 2 weeks every 3 weeks (Schedule 2/1), starting on Day 2. Sunitinib starting dose will be 37.5 mg once daily.

Self-administration of sunitinib capsules will take place on an outpatient basis. Capsules should be taken once daily without regard to meals.

Patients will be monitored for toxicity, and the sunitinib dose may be adjusted according to individual patient tolerance (see table below).

Table: Dose levels for dose reduction

Dose Level	SUNITINIB (Schedule 2/1)
0	37.5 mg
-1	25 mg

The dose may be reduced to 25 mg daily in cycles ≥2 depending on individual tolerability as reported (see table below). The 25 mg/day will be the minimum dose acceptable. In subsequent cycles (cycles >2), intrapatient re-escalation of sunitinib back to the previous dose level is at the discretion of the investigator and considering the patient's clinical status. The patient should be discontinued from the study if the current dose level is 25 mg and toxicity guidelines indicate a further dose reduction is necessary. Maximum acceptable interval between last and next sunitinib administrations due to persistent toxicity attributable to sunitinib or delay in docetaxel administration should be 3 weeks. In case of an interval >3 weeks, treatment continuation is at the discretion of the Investigator.

Table: Dose Modifications for Sunitinib related Toxicities for a New Cycle Based on Worst Toxicity Observed in the Prior Cycle

Toxicity (NCI CTC Grade, Version 3.0)			
**Grade 1**	**Grade 2**	**Grade 3**	**Grade 4**
**Hematologic toxicities**			
**Continue at the same dose level**	**Continue at the same dose level**	**Withhold dose until toxicity is grade ≤2 or has returned to baseline, then resume treatment at the same dose level or reduce the dose by 1 level at the discretion of the investigator (1)**	**Withhold dose until toxicity is grade ≤2 or has returned to baseline, then reduce the dose by 1 level and resume treatment (1)**
**Non-hematologic toxicities**			
**Continue at the same dose level**	**Continue at the same dose level**	**Withhold dose until toxicity is grade ≤1 or has returned to baseline, then resume treatment at the same dose level or reduce the dose by 1 level at the discretion of the investigator (2)**	**Withhold dose until toxicity is grade ≤1 or has returned to baseline, then reduce the dose by 1 level and resume treatment, or discontinue at the discretion of the investigator (2)**

(1) In case of **complicated severe neutropenia** (ie, febrile neutropenia, neutropenic infection, and prolonged grade 4 neutropenia) dose reduction of sunitinib will be considered only if neutropenia cannot be attributed to docetaxel or if sunitinib contribution to the event cannot be ruled out [median time to nadir of docetaxel is 7 days; time to nadir of sunitinib may approach day 14-21 with schedule 2/1].

In case of **grade 3 or 4 lymphopenia** dose may be maintained without interruption.

(2) In case of **grade 4 hyperuricemia** or **grade 3 hypophosphatemia** without clinical symptoms, the dose may be maintained at the discretion of the investigator. In case of **grade 3 or 4 hyperamylasemia or hyperlipasemia** without clinical signs of pancreatitis, the dose may be maintained at the discretion of the investigator.

In case of **nausea, vomiting, or diarrhea** reduce the dose only if the events persist at grade 3 or 4 despite maximal medical therapy.

10 ADVERSE EVENTS (AEs) AND SERIOUS ADVERSE EVENTS (SAEs)

10.1 DEFINITION OF ADVERSE EVENT, ADVERSE DRUG REACTION AND SERIOUS ADVERSE EVENT

10.1.1 Adverse events (or adverse experience) (AE):

An AE is any untoward medical occurrence in a patient or clinical investigation subject administered a pharmaceutical product and which does not necessarily have a causal relationship with this treatment.

An AE can therefore be any unfavorable and unintended sign (including an abnormal laboratory finding, for example), symptom, or disease temporally associated with the use of a medicinal product, whether or not considered related to the medicinal product.

Due to regulatory requirements, events occurring during pre- and post-treatment periods should also be designated as AEs. Therefore, safety surveillance (reporting of AEs) commences at the time when the patient is enrolled into the study (date of signature of the informed consent to study participation) until the End of Study Visit has been performed. Therefore events occurring in the period between signature of informed consent and beginning of the study drug administration are to be designated as AEs. This procedure complies with requirements by some authorities.

10.1.2 Adverse drug reaction (ADR):

All noxious and unintended responses to a medicinal product related to any dose should be considered adverse drug reactions (ADRs). The phrase "responses to a medicinal product" means that a causal relationship between a medicinal product and an AE is at least a reasonable possibility i.e. the relationship cannot be ruled out.

10.1.3 Serious adverse event or reaction/experience (SAE)**:**

A serious AE (experience) or reaction is any untoward medical occurrence that at any dose:

- Results in death
- Is life-threatening

  NOTE: The term ”life-threatening” in the definition of ”serious” refers to an event in which the patient was at risk of death at the time of the event; it does not refer to an event which hypothetically might have caused death if it were more severe.

- Requires in-patient hospitalization or prolongation of existing hospitalization
- Results in persistent or significant disability/incapacity
- Is a congenital anomaly/birth defect or
- Is an important medical event

Medical and scientific judgement should be exercised in deciding whether expedited reporting is appropriate in cases of important medical events that may not be immediately life-threatening or result in death or hospitalization but may jeopardize the patient or may require intervention to prevent one of the other outcomes listed in the definition above. These should also usually be considered serious.

Examples of such events are intensive treatment in an emergency room, or at home for allergic bronchospasm; blood dyscrasias or convulsions that do not result in hospitalization; or development of drug dependency or drug abuse, or malignant tumors when they are histologically different from the primary tumor.

- **Events not to be treated as SAEs**

Progression of disease is not to be regarded as a SAE.

Due to the seriousness of the disease in this study, certain conditions defined as SAEs will be excluded from expedited reporting on a SAE report Form, i.e.:

- Elective hospitalization and surgery for treatment of disease
- Elective hospitalization to simplify treatment or study procedures

10.2 Methods of recording and assessing adverse events

All AEs must be documented in the appropriate section of the CRF. For SAEs a SAE report Form (initial or follow up) must be completed in addition.

The following aspects must be recorded for each event in the CRF:

- A description of the AE in medical terms, not as reported by the patient;
- The date of onset (start date)
- Time of onset in case event started at the day of sunitinib administration (start time)
- The date of recovery (stop date)
- Time of recovery in case event stopped at the day of sunitinib administration (stop time)
- The grade as assessed by the investigator according to the definitions in NCI-CTC, Version 3.0

  Grade 1=mild

  Grade 2=moderate

  Grade 3=severe

  Grade 4=life-threatening or disabling

  Grade 5=death related to AE
- The causal relationship to sunitinib or docetaxel as assessed by the investigator; the decisive factor in the documentation is the temporal relation between the AE and the study drug
- Seriousness: yes or no

In case of SAEs it must be indicated whether the SAE is the leading event (primary medical reason).

If in any one patient the same AE occurs on several occasions, then the AE in question must be documented and assessed anew each time.

10.2.1 Procedure for reporting serious adverse events

In the event of the occurrence of any clinical AE or abnormal laboratory test value that is serious or medically important during the course of the study or the post-treatment period, irrespective of the treatment received by the patient, the investigator is obliged to immediately inform the sponsor either by telephone, by fax or by e-mail

The SAE report Form and the respective completion guidelines are described in a separate document.
For names, addresses and telephone see SAE report Form.

Following a report by phone, written information has to be sent by fax or e-mail. For written reports, the SAE report Form (for an “initial” SAE or for “follow-up” information on a previous SAE) must be used.

As a principle, the SAE must be documented and medically assessed by the investigator and the outcome described in the AE section of the CRF.
Additional information will be requested, if necessary, by the sponsor. This is to ensure that the initial reporting of SAEs is made to regulatory authorities within the requested timeframe.

The sponsor will distribute a CIOMS-I form of Suspected Unexpected Serious Adverse Reactions (SUSARs) according to the valid investigator brochure for sunitinib and the valid product information of other drugs. The sponsor is solely responsible for sending the reports on SUSARs to all participating investigators, to Regulatory agencies and Ethics Committees concerned in accordance with international and local laws and regulations as well as ICH/GCP guidelines
With respect to sunitinib, the sponsor shall immediately inform Merck of any sunitinib SUSAR, which has been a submitted expeditedly to competent authorities and ethics committee. Sunitinib SUSARs represent Serious Adverse Events related to sunitinib (=Adverse Reactions), considered “unexpected” with regard to the valid investigator brochure for sunitinib.

For a follow-up report to the authorities, the sponsor may collect further information for a detailed description and a final evaluation of the case, including copies of hospital reports, autopsy reports, or other relevant documents.

The sponsor must notify the Ethics Committee (EC) / Institutional Review Board (IRB) and other investigators, if appropriate, in accordance with international and local laws and regulations.

10.3 Monitoring of patients with adverse events

Any AE that occurs in the course of a clinical study must be monitored and followed up until the End of Study Visit. In addition SAEs must be reported via an SAE report Form directly to the sponsor. It is the responsibility of the investigator that any necessary additional therapeutic measures and follow-up procedures are performed.

11 STATISTICAL CONSIDERATIONS

11.1 Analysis of biomarker

Analyses of biomarker will be performed considering all observed data. If not otherwise specified missing data will not be replaced. Continuous variables will be summarized using descriptive statistics i.e., mean, median, geometric mean, standard deviation, standard error of the mean, minimum, maximum. Mean differences between measurements at different time points will be computed for continuous variables with two-sided 95% confidence intervals if appropriate. Qualitative variables will be summarized by means of counts and percentages.

11.2 Analysis of safety

The adverse events will be coded based on the NCI-CTC version 3.0 by the investigator. The frequencies of the adverse event by group terms will be computed. Incidence and type of adverse events will be summarized. Adverse events that are reported as "possible", "probable" or "definite" related to study medications will be considered related to study drug; missing classifications concerning study drug relationship will be considered as related to the study

drug. The frequencies of drug-related adverse events will also be computed. The adverse event frequencies will also be reported by severity grade.
Laboratory values will be evaluated using mean values and standard deviations. The number of patients who had values outside normal ranges will be given and values considered clinically relevant by the investigator will be listed.

11.3 Sample-size

The sample size is considered sufficient for PD-analysis. There is no statistical justification for the sample size.

12 ETHICAL AND REGULATORY ASPECTS

12.1 Risk/Benefit assessment

No standard treatment or concomitant medication will be withheld from any patient. All HRPC patients will receive first choice standard chemotherapy with docetaxel. Sunitinib, which has already been approved for metastatic renal cell cancer and GIST and was tolerated in a Phase I in combination with docetaxel, will be administered orally as an anti-angiogenic drug for improving tumor response to docetaxel. Anticipated and particularly closely monitored side effects include primarily hematological parameters. The potential benefits appear to clearly outweigh the risks.

12.2 Responsibilities of the Investigator

The investigator shall be responsible for ensuring that the clinical study is performed in accordance with the protocol, the Declaration of Helsinki as well as with the Note for Guidance on Good Clinical Practice (CPMP/ICH/135/95) approved July 17, 1996 and applicable regulatory requirements. These documents state that the informed consent of the patients is an essential precondition for participation in the clinical study.

12.3 Subject information and informed consent

An unconditional prerequisite for participation of a patient in the clinical study is his/her written informed consent after having been informed about all relevant aspects of the study. The informed consent form will be created and handled according to the ICH Note for Guidance on Good Clinical Practice dated July 17, 1996.

12.4 Ethics Committee or Institutional Review Board

Prior to commencement of the study, the study protocol will be submitted together with its associated documents (patient information, consent form, investigator's brochure) to the relevant EC for their favorable opinion. The favorable opinion/approval of the Ethics Committee will be filed in the investigator study file and a copy in the study master file at the sponsor.

The study will only commence following provision of a written favorable opinion. Documentation of the date of the meeting, constitution of the committee and voting members present at the meeting will be requested by the sponsor, who will also require written evidence that clearly identifies the trial, protocol version, and consent documents reviewed. Where possible, copies of the minutes of the meeting in respect of the submitted protocol will be obtained.

Any amendments to the protocol will be submitted to the EC and they will be informed about SAEs in accordance with national and/or local requirements.

12.5 Notification to authorities

The study protocol and any applicable documentation will be notified to authorities in accordance with local regulations.

13 ADMINISTRATIVE CONSIDERATIONS

13.1 Case report form handling

Case report forms (CRFs) will be specifically created for this trial and will be used for documentation. It is critical to adhere to the schedule of visits prescribed in the protocol for all randomized patients. The forms (except for serious adverse events) will be collected by the study monitor. The main objective is to obtain those data required by the study protocol in a complete, accurate, legible and timely fashion. The data in the CRF should be consistent with the relevant source documents.

The data recorded in the course of this study must be documented in the CRF and/or the “SAE report form” and must be forwarded to the sponsor. They shall then be processed, evaluated, and stored anonymously in accordance with the data-protection regulations. The investigator must ensure that the CRFs forwarded to the sponsor and any other associated documents contain no mention of any patient names.

The CRFs must be filled in completely and legibly (with either black or blue ballpoint pen, acceptable for use on official documents). Any amendments and corrections necessary must be undertaken and countersigned by the investigator, stating the date of the amendment/correction. Errors must remain legible and may not be deleted with correction aids (e.g. Tipp-Ex®). The investigator must state his/her reasons for the correction of important data.

In the case of missing data/remarks, the entry spaces provided for in the case report form should be cancelled out so as to avoid unnecessary follow-up inquiries.

13.2 Source data and subject files

The investigator must keep a written or electronic subject file for every subject participating in the clinical study. In this subject file, the available demographic and medical information of a subject has to be documented, in particular the following: name, date of birth, sex, height, weight, subject history, concomitant diseases and concomitant drug (including changes during

the study), statement of entry into the study, study identification, subject number, the date of informed consents, all study visit dates, predefined performed examinations and clinical findings, observed adverse events (if applicable), and reason for withdrawal from the study if applicable. It should be possible to verify the inclusion and exclusion criteria for the study from the available data in this file.

It must be possible to identify each subject by using this patient file.

Additionally, any other documents with source data, especially original printouts of data that were generated by technical equipment have to be filed. All these documents have to bear at least subject identification and the printing date printed by the recording device to indicate to which subject and to which study procedure the document belongs. The medical evaluation of such records should be documented as necessary and signed/dated by the investigator.

Computerized subject files will be printed whenever source data verification is performed by the monitor. Printouts must be signed and dated by the investigator, countersigned by the monitor and kept in a safe place.

Data that has to be recorded directly on the CRFs (i.e. no prior written or electronic record of data) are considered to be source data.

13.3 Investigator site file and archiving

The investigator will be provided with an ISF by the sponsor at the start of the study. This file contains all relevant documents necessary for the conduct of the study. This file must be safely archived after termination of the study.

It is the responsibility of the investigator to ensure that the patient-identification sheets are stored for at least 15 years beyond the end of the clinical study. All original patient files must be stored for the longest possible time permitted by the regulations at the hospital, research institute, or practice in question. If archiving can no longer be maintained at the site, the investigator will notify the sponsor.

13.4 Monitoring, quality assurance and inspection by authorities

This study is to be conducted in accordance with the Note for Guidance on Good Clinical Practice. The appointed clinical monitor will arrange regular visits to the study center(s) on a regular basis to check progress with the study and to collect completed CRFs.

During monitoring visits the monitors will:

- Help resolve any problems.
- Examine all CRFs for omission of data, compliance and possible adverse events.
- Discuss inconsistencies in the trial data.
- Ensure that all trial materials are correctly stored and dispensed.
- Check adherence to the obligations of the investigator.
- Review consent forms, in particular the date of consent and signature.
- Perform Source Data Verification as described below.

In line with ICH GCP guidelines monitoring will include verification of data entered in the CRFs against original patient records. This verification will be performed by direct access to the original patient records and the company guarantees that patient confidentiality will be respected at all times. Participation in this study will be taken as agreement to permit direct source data verification. Data generated at baseline and at on-study visits are verified against source data only in case the patient enters the study.

In addition the representatives of a Quality Assurance Unit will be permitted to inspect the study documents (study protocol, case report forms, study medication, original medical records/files). All patient data shall be treated confidentially.

In the course of the clinical study, the CRFs shall be forwarded to the sponsor after completion of the individual sections (e.g. visits) of the study.

The study protocol, each step of the data-recording procedure, and the handling of the data as well as the study report may be subject to an independent Clinical Quality Assurance review. Audits can be conducted to assure the validity of the study data.

13.5 Changes to the study protocol

Changes to, or formal clarifications of, the study protocol must be documented in writing.

Major changes to the protocol will be described in a "Protocol Amendment". It will be submitted to the relevant Ethics Committee(s)/Institutional Review Board(s) and to authorities where required. Approval/favorable opinion from the relevant Ethics Committee(s)/Institutional Review Board(s) and from authorities if applicable will be required prior to implementation of the amendment.

13.6 Study report and publication

After conclusion of the study, an integrated clinical and statistical study report shall be written by the Sponsor and coordinating investigator. Any publications of the results, either in part or in total (abstracts in journals or newspapers, oral presentations, etc.) by investigators or their representatives will require pre-submission review by the Sponsor.

14 REFERENCES

Reference List

1 Cooperberg,M.R., Moul,J.W. and Carroll,P.R. The changing face of prostate cancer, J.Clin.Oncol., *23:* 8146-8151, 2005.

2 Tannock,I.F., de,W.R., Berry,W.R., Horti,J., Pluzanska,A., Chi,K.N., Oudard,S., Theodore,C., James,N.D., Turesson,I., Rosenthal,M.A. and Eisenberger,M.A. Docetaxel plus prednisone or mitoxantrone plus prednisone for advanced prostate cancer, N.Engl.J.Med., *351:* 1502-1512, 2004.

3 Petrylak,D.P., Tangen,C.M., Hussain,M.H., Lara,P.N., Jr., Jones,J.A., Taplin,M.E., Burch,P.A., Berry,D., Moinpour,C., Kohli,M., Benson,M.C., Small,E.J., Raghavan,D. and Crawford,E.D. Docetaxel and estramustine compared with mitoxantrone and prednisone for advanced refractory prostate cancer, N.Engl.J.Med., *351:* 1513-1520, 2004.

4 Bertolini,F., Shaked,Y., Mancuso,P. and Kerbel,R.S. The multifaceted circulating endothelial cell in cancer: towards marker and target identification, Nat Rev Cancer, *6:* 835-845, 2006.

5 Shaked,Y., Emmenegger,U., Man,S., Cervi,D., Bertolini,F., Ben-David,Y. and Kerbel,R.S. Optimal biologic dose of metronomic chemotherapy regimens is associated with maximum antiangiogenic activity, Blood, *106:* 3058-3061, 2005.

6 Furstenberger,G., von,M.R., Lucas,R., Thurlimann,B., Senn,H.J., Hamacher,J. and Boneberg,E.M. Circulating endothelial cells and angiogenic serum factors during neoadjuvant chemotherapy of primary breast cancer, Br.J.Cancer, *94:* 524-531, 2006.

7 Rafii,S. Circulating endothelial precursors: mystery, reality, and promise, J.Clin.Invest, *105:* 17-19, 2000.

8 Ribatti,D. The involvement of endothelial progenitor cells in tumor angiogenesis, J.Cell Mol.Med., *8:* 294-300, 2004.

9 Guerin,O., Formento,P., Lo,N.C., Hofman,P., Fischel,J.L., Etienne-Grimaldi,M.C., Merlano,M., Ferrero,J.M. and Milano,G. Supra-additive antitumor effect of sunitinib malate (SU11248, Sutent((R))) combined with docetaxel. A new therapeutic perspective in hormone refractory prostate cancer, J.Cancer Res.Clin.Oncol., 2007.

10 Mancuso,P., Colleoni,M., Calleri,A., Orlando,L., Maisonneuve,P., Pruneri,G., Agliano,A., Goldhirsch,A., Shaked,Y., Kerbel,R.S. and Bertolini,F. Circulating endothelial-cell kinetics and viability predict survival in breast cancer patients receiving metronomic chemotherapy, Blood, *108:* 452-459, 2006.

11 Hotchkiss,K.A., Ashton,A.W., Mahmood,R., Russell,R.G., Sparano,J.A. and Schwartz,E.L. Inhibition of endothelial cell function in vitro and angiogenesis in vivo by

docetaxel (Taxotere): association with impaired repositioning of the microtubule organizing center, Mol.Cancer Ther., *1:* 1191-1200, 2002.

12 Lennernas,B., Albertsson,P., Lennernas,H. and Norrby,K. Chemotherapy and antiangiogenesis--drug-specific, dose-related effects, Acta Oncol., *42:* 294-303, 2003.

13 Shaked,Y., Ciarrocchi,A., Franco,M., Lee,C.R., Man,S., Cheung,A.M., Hicklin,D.J., Chaplin,D., Foster,F.S., Benezra,R. and Kerbel,R.S. Therapy-induced acute recruitment of circulating endothelial progenitor cells to tumors, Science, *313:* 1785-1787, 2006.

14 Bruno,R., Hille,D., Riva,A., Vivier,N., ten Bokkel Huinnink,W.W., van Oosterom,A.T., Kaye,S.B., Verweij,J., Fossella,F.V., Valero,V., Rigas,J.R., Seidman,A.D., Chevallier,B., Fumoleau,P., Burris,H.A., Ravdin,P.M. and Sheiner,L.B. Population pharmacokinetics/pharmacodynamics of docetaxel in phase II studies in patients with cancer, J.Clin.Oncol., *16:* 187-196, 1998.

Attachment I

Flow Chart

	Baseline / Prior to Study Treatment		Study Treatment					
	28 days before study drug administration	14 days before study drug administration	Every Day 1 of each cycle (Phase 1 and 2)	Day 3, 8, and 15 of each cycle (Phase 1)	Day 15 (Phase 2)	After cycle 4 (if clinically indicated)	Treatment holidays (every 3 weeks)	End of study visit
Informed consent	X							
Imaging tumor assessment (CT or MRI scan)	X					X		
Bone scan	X					X		
Chest X-ray (if no chest CT or MRI done)	X					X		
Echocardiography or MUGA scan	X					X		X
ECG			X					X
Medical history		X						
Inclusion/Exclusion criteria		X						
Concomitant medication		X	X				X	X
Weight		X	X					
Height		X						
Vital signs		X	X					X
Physical exam incl WHO Performance		X	X			X		X
Blood sampling for clinical chemistry		X	X	only d8	X			X
Blood sampling for hematology		X	X	X	X			X
Blood sampling for PSA	X		X			X	X	X
Blood sampling for testosterone	X							
Blood sampling pharmacodynamics		X	X	X	X	X		X
(Serious) Adverse Events		X	X			X	X	X

Attachment II

WHO Performance Status Criteria

Grade	Performance scale
0	Able to carry out all normal activity without restriction.
1	Restricted in physically strenuous activity but ambulatory and able to carry out light work.
2	Ambulatory and capable of all self-care but unable to carry out any work; up and about more than 50% of waking hours.
3	Capable of only limited self-care; confined to bed or chair more than 50% of waking hours.
4	Completely disabled; cannot carry on any self-care; totally confined to bed or chair.

Attachment III

Protocol Signatures

I confirm that I have read this protocol, I understand it, and I will work according to this protocol and to the principles of good clinical practice, or the applicable laws and regulations of the country of the study site for which I am responsible.

______________________________ ______________________________

Investigator Signature Date

____

Investigator name

____

Facility address

______________________________ ______________________________

Signature Sponsor Date

____

Name Sponsor representative
